# Nucleus accumbens D1/D2 circuits control opioid withdrawal symptoms in mice

**DOI:** 10.1172/JCI163266

**Published:** 2023-09-15

**Authors:** Yongsheng Zhu, Kejia Wang, Tengfei Ma, Yuanyuan Ji, Yin Lou, Xiaoyu Fu, Ye Lu, Yige Liu, Wei Dang, Qian Zhang, Fangyuan Yin, Kena Wang, Bing Yu, Hongbo Zhang, Jianghua Lai, Yunpeng Wang

**Affiliations:** 1 College of Forensic Science, Key Laboratory of National Health Commission for Forensic Science, National Biosafety Evidence Foundation, Xi’an Jiaotong University, Xi’an, China.; 2 Fujian Provincial Key Laboratory of Organ and Tissue Regeneration, Xiamen Key Laboratory of Regeneration Medicine, Organ Transplantation Institute of Xiamen University, School of Medicine, Xiamen University, Xiamen, China.; 3 School of Pharmacy, Nanjing Medical University, Nanjing, China.; 4 The Sixth Ward, Xi’an Mental Health Center, Xi’an, China.; 5 Department of Biochemistry and Molecular Biology, School of Basic Medical Sciences, Ningxia Medical University, Yinchuan, China.; 6 Department of Psychiatry and Center for Brain Science, The First Affiliated Hospital of Xi’an Jiaotong University, Xi’an, China.; 7 Shaanxi Belt and Road Joint Laboratory of Precision Medicine in Psychiatry, The First Affiliated Hospital of Xi’an Jiaotong University, Xi’an, China.

**Keywords:** Neuroscience, Addiction, Depression, Mouse models

## Abstract

The nucleus accumbens (NAc) is the most promising target for drug use disorder treatment. Deep brain stimulation (DBS) of NAc is effective for drug use disorder treatment. However, the mechanisms by which DBS produces its therapeutic effects remain enigmatic. Here, we define a behavioral cutoff criterion to distinguish depressive-like behaviors and non-depressive-like behaviors in mice after morphine withdrawal. We identified a basolateral amygdala (BLA) to NAc D1 medium spiny neuron (MSN) pathway that controls depressive-like behaviors after morphine withdrawal. Furthermore, the paraventricular nucleus of thalamus (PVT) to NAc D2 MSN pathway controls naloxone-induced acute withdrawal symptoms. Optogenetically induced long-term potentiation with κ-opioid receptor (KOR) antagonism enhanced BLA to NAc D1 MSN signaling and also altered the excitation/inhibition balance of NAc D2 MSN signaling. We also verified that a new 50 Hz DBS protocol reversed morphine withdrawal–evoked abnormal plasticity in NAc. Importantly, this refined DBS treatment effectively alleviated naloxone-induced withdrawal symptoms and depressive-like behaviors and prevented stress-induced reinstatement. Taken together, the results demonstrated that input- and cell type–specific synaptic plasticity underlies morphine withdrawal, which may lead to novel targets for the treatment of opioid use disorder.

## Graphical abstract

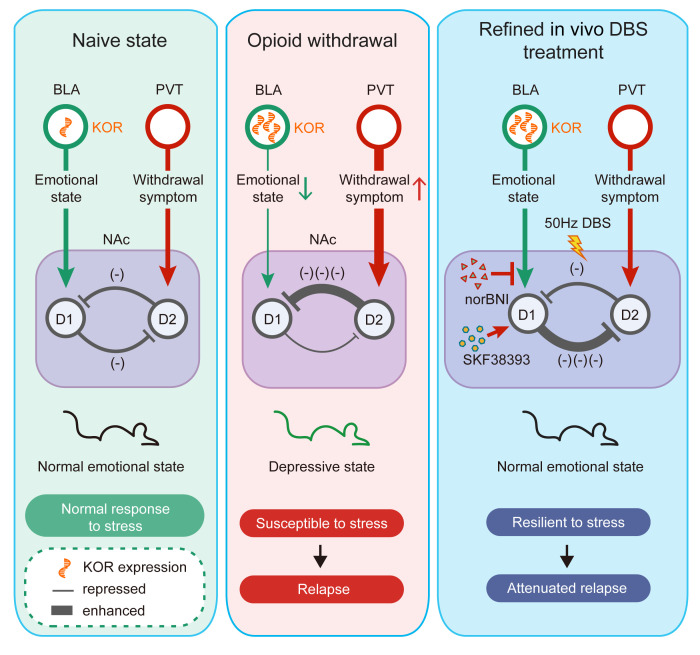

## Introduction

Opioid drugs are powerful analgesics, but they are also highly addictive due to the elicitation of powerful euphoria. Opioid use disorder depends not only on its positive reinforcement effects, but also on the negative consequences of withdrawal. Early withdrawal symptoms in patients with opioid use disorder include trembling, muscle aches and pains, insomnia, spontaneous ejaculation, etc. (1, 2). Furthermore, psychological symptoms and drug craving increase in intensity during the withdrawal period (3, 4). Depression is a frequent neuropsychiatric comorbidity in patients with opioid use disorder (5). Heroin use disorder patients with depression have higher rates of attempted suicide and poor long-term treatment outcomes (6, 7).

The nucleus accumbens (NAc) is a primary site that orchestrates both the reward and the aversion processes. NAc dysfunction is directly involved in withdrawal symptoms and depressive-like behaviors (8, 9). Approximately 95% of NAc neurons are medium spiny neurons (MSNs), which can be further divided into dopamine D1 and D2 receptor–expressing neurons (10, 11). D1 and D2 MSNs have opposite activities in addictive drug–associated behaviors (12). Considerable progress has been made in characterizing the neural circuits that underlie key components of drug-seeking behaviors. Several limbic, cortical, and thalamic regions, including the basolateral amygdala (BLA), the ventral hippocampus (vHip), the medial prefrontal cortex (mPFC), and the paraventricular nucleus of the thalamus (PVT), send projections to the NAc. Specifically, excitatory afferents that arise from the BLA, vHip, and mPFC regulate cue-associated reward-seeking behaviors (13, 14), while inputs of the PVT to the NAc are required for the manifestation of aversion to opioid withdrawal (8). However, little is known about how D1 and D2 MSNs represent somatic or emotional information arising from these diffuse nuclei in the context of opioid withdrawal.

The κ-opioid receptor (KOR) regulates neural systems associated with anhedonia and aversion and mediates negative affective states associated with psychiatric disorders. KOR antagonists have been recommended as novel antidepressants (15). The overwhelming evidence indicates that KOR regulates neuronal excitation in NAc (10). This regulation suggests an important function of KOR within NAc in opioid withdrawal–related behaviors (16–18). In rodents, KOR antagonism in NAc prevented stress-induced reinstatement of cocaine seeking (19) and attenuated increased motivation for heroin intake over time (20). Although KOR signaling in NAc is involved in the regulation of opioid use disorder and antidepressant-like effects, our understanding of how distinct afferents to NAc with KOR are related to stress-triggered opioid reinstatement remains poor.

In particular, preclinical studies and case studies have reported that deep brain stimulation (DBS) of NAc is successful for drug use disorder treatment (21). Although high-frequency DBS (>100 Hz) is effective, its effects are generally transient, with symptoms recurring after cessation of stimulation (22). Furthermore, high-frequency DBS is unlikely to reverse the neuroplastic changes that occur in response to drug use disorder (23). Therefore, it is necessary to refine DBS treatment based on pathway- and cell-specific circuits to alleviate early (physical) and chronic (psychological) withdrawal symptoms and prevent relapse.

Here, we systematically assessed naloxone-induced withdrawal symptoms and emotional states in mice after protracted withdrawal using quantitative and qualitative analyses. We observed input- and cell type–specific alterations in synaptic plasticity in BLA→NAc^D1^ and PVT→NAc^D2^ pathways, which control depressive-like behaviors and naloxone-induced withdrawal symptoms, respectively. Especially, refined DBS treatment with KOR blockade markedly restored synaptic deficits and prevented naloxone-induced withdrawal symptoms, depressive-like behaviors, and stress-induced reinstatement in mice.

## Results

### Morphine-treated mice show depressive-like behaviors after a protracted withdrawal period.

**Figure 1:**
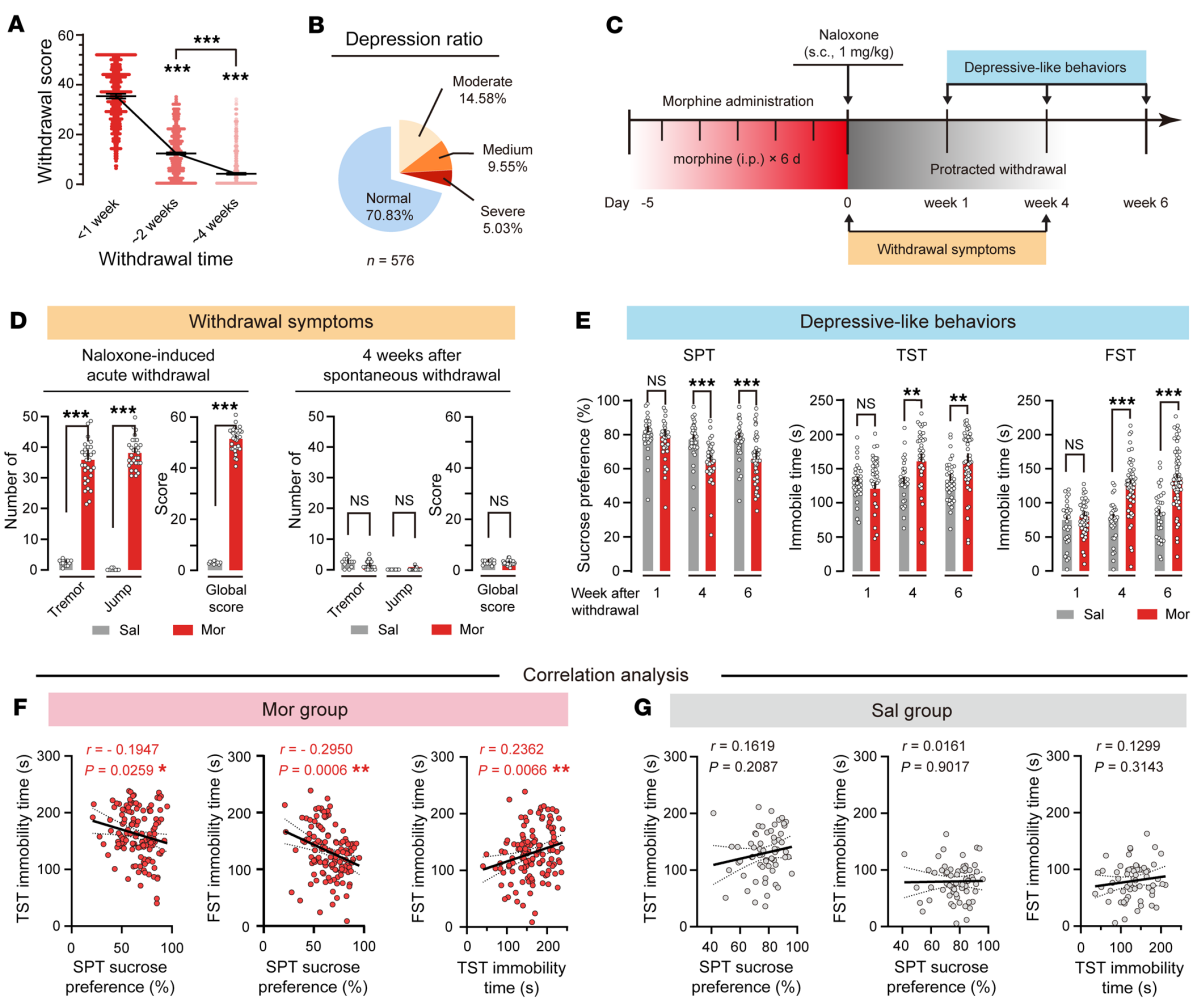
Mice treated repeatedly with morphine show depressive-like behaviors during a protracted withdrawal period. (**A**) Withdrawal score within 4 weeks of drug withdrawal in heroin users. *n* = 576 per group. ***P < 0.0001, by repeated-measures (RM) 1-way ANOVA followed by Šidák’s test. (**B**) Depressive ratio of heroin users. (**C**) Flowchart of withdrawal symptoms and behavioral tests. (**D**) Withdrawal symptoms. *n* = 30 per group. ***P < 0.0001, by Mann-Whitney *U* test. (**E**) Depressive-like behaviors appeared 4 weeks after morphine withdrawal. *n* = 62 saline (Sal) mice, and *n* = 131 morphine (Mor) mice. **P < 0.01, ***P < 0.0001, by mixed RM 2-way ANOVA followed by Šidák’s test. (**F** and **G**) Correlation analysis between SPT sucrose preference, TST immobility time, and FST immobility time in Mor group and Sal group. *P < 0.05, **P < 0.01, by Pearson’s correlation test. Data are presented as mean ± SEM.

According to our survey, physical disturbance in long-term heroin users usually disappeared within 4 weeks after withdrawal (Figure 1A). Moderate to severe depression symptoms appeared in 29.2% of heroin users after 4 weeks of withdrawal (*n* = 169/576; Figure 1B). This frequency was significantly higher than 0.54% of the control population (24). Because depression in opioid users is significantly associated with a high rate of relapse (6, 7), we examined somatic and emotional changes after morphine withdrawal. Mice daily received 2 intraperitoneal (i.p.) injections of escalating doses of morphine from 10 to 50 mg/kg for 6 days (Figure 1C). To confirm physical dependence, mice were treated with a single dose of naloxone (1 mg/kg, s.c.) 2 hours after the last injection of morphine. As expected, naloxone induced strong acute withdrawal symptoms in morphine-treated mice (Figure 1D, left). However, these symptoms were not observed 4 weeks after spontaneous withdrawal (Figure 1D, right; also see Supplemental Table 2). To assess emotional changes after morphine withdrawal, we performed a series of depressive-like behavior tests, including the sucrose preference test (SPT), tail suspension test (TST), and forced swim test (FST), at 1, 4, and 6 weeks after spontaneous withdrawal. Interestingly, depressive-like behaviors gradually appeared after 4 weeks of withdrawal in morphine-treated mice (Figure 1E), a time scale similar to that observed for heroin users. The magnitudes of depressive-like behaviors were highly correlated with each other after protracted withdrawal. Specifically, the lower the sucrose preference in SPT, measured 4 weeks after cessation of morphine administration, the longer the immobile time in TST and FST (Figure 1F). There was no correlation between 3 depressive measures in saline mice (Figure 1G). These results validated that sucrose preference in SPT and immobility time in TST and FST are suitable and efficient measures for depressive-like behaviors in mice after protracted morphine withdrawal. Anxiety and body weight were also assessed in mice during 4 weeks of morphine withdrawal (Supplemental Figure 1, A and B; supplemental material available online with this article; https://doi.org/10.1172/JCI163266DS1). Anxiety-like behaviors were observed only 1 week after spontaneous withdrawal (Supplemental Figure 1, C and D). The correlation was not observed between anxiety- and depressive-like behaviors after 4 weeks of withdrawal (Supplemental Figure 1, E–H).

### A subpopulation of morphine withdrawal mice with depressive-like phenotypes are vulnerable to stress-induced CPP reinstatement.

**Figure 2:**
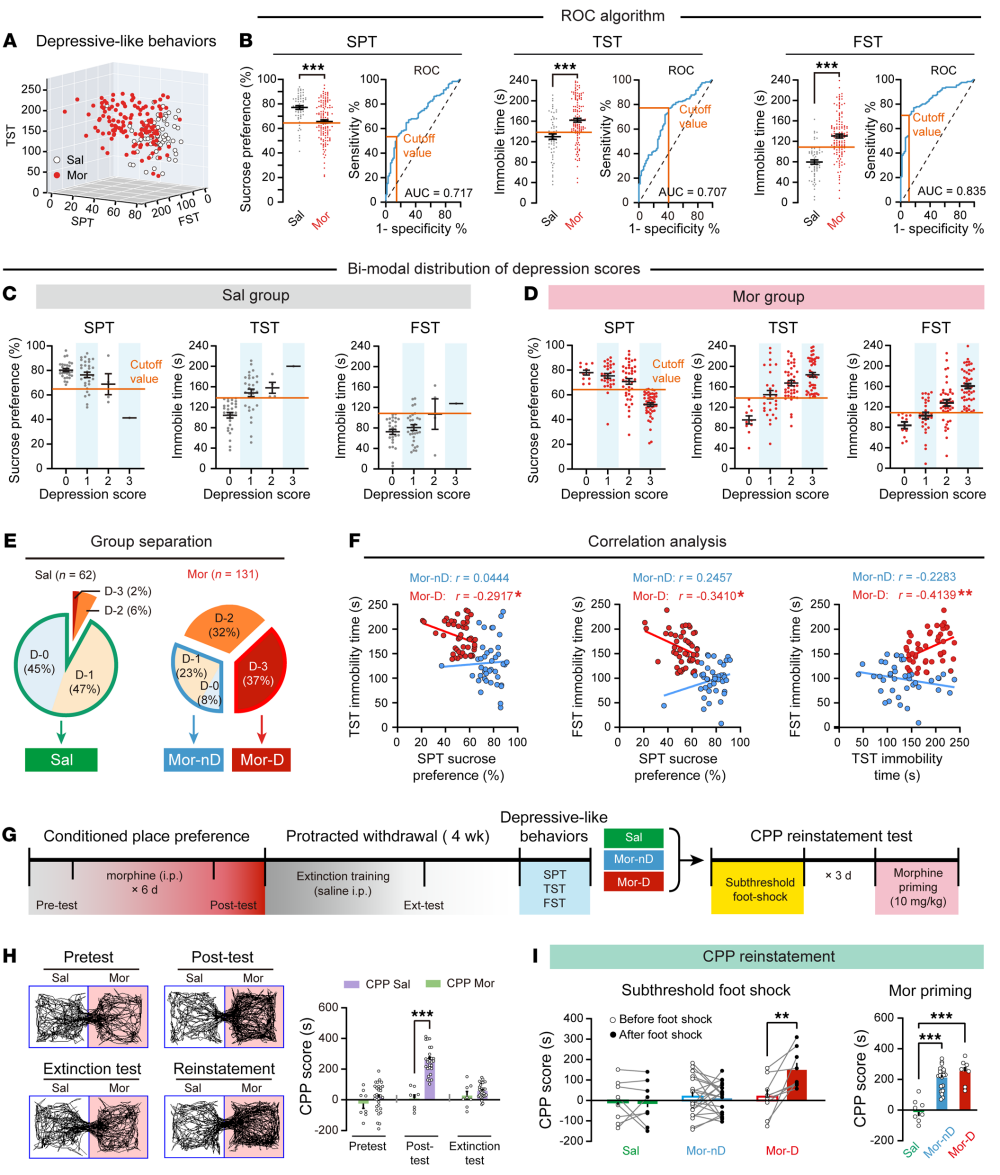
A subpopulation of morphine withdrawal mice with depressive-like phenotypes are vulnerable to stress-induced CPP reinstatement. (**A**) Three-dimensional plot showing the variability in depressive-like behaviors of SPT, TST, and FST. (**B**) ROC algorithm to establish cutoff criteria based on SPT, TST, and FST. Orange line, cutoff value based on the maximum Youden index. AUC, area under the curve (0–1). *n* = 62 Sal, and *n* = 131 Mor. ***P < 0.0001, by unpaired Student’s *t* test. (**C** and **D**) Bimodal distribution of depression scores. Sal and Mor mice were considered positive for behavioral criteria 0 to 3 according to the cutoff value (orange line). (**E**) Separation of groups according to depression score. Mor mice that met all 3 positive criteria (D-3) were classified as the depressive-like phenotype (Mor-D). Mor mice with a D-0 or D-1 were classified as the non-depressive-like phenotype (Mor-nD). The Sal group only included mice with D-0 or D-1. (**F**) Correlation analysis between SPT sucrose preference, TST immobility time, and FST immobility time in Mor-nD and Mor-D group. *P < 0.05, **P < 0.01, by Pearson’s correlation test. (**G**) Flowchart of the CPP reinstatement test. (**H**) Left: Example traces of morphine CPP tests. The red chamber indicates the morphine-paired chamber. Right: CPP scores in pretest, post-test, and extinction test. (**I**) CPP reinstatement test. Left: CPP score before and after foot shock in different groups. *n* = 8 Sal, *n* = 21 Mor-nD, and *n* = 9 Mor-D. **P < 0.01, by mixed RM 2-way ANOVA followed by Šidák’s test. Right: A priming dose of morphine evoked CPP reinstatement in different groups. ***P < 0.0001, by 1-way ANOVA followed by Tukey’s test. Data are presented as mean ± SEM.

Clinical studies of depression in heroin users distinguish affected and unaffected individuals based on the severity of symptoms. We found a high variability in depressive-like behaviors after protracted morphine withdrawal (Figure 2A). Currently, there are no clear behavioral cutoff criteria to define depressive-like phenotypes in a rodent model of opioid withdrawal. We used a receiver operating characteristic (ROC) algorithm to establish cutoff criteria based on depressive-like behaviors in mice. The areas under curves (AUCs) for 3 measures were greater than 0.7 (Figure 2B). Specifically, cutoff criteria were determined according to the Youden index (25), which allowed us to make an unbiased decision whether an individual animal was positive for a given behavioral phenotype. The cutoff values that had a combination of the highest true positive and lowest false positive rates were 64.78% for the sucrose preference of SPT, 138.04 seconds for the immobile time of TST, and 108.67 seconds for the immobile time of FST. We separated saline- or morphine-treated mice into subgroups according to the number of positive criteria met; then we assigned mice a score of depressive-like behaviors from 0 to 3 (D score). Strikingly, both saline- and morphine-treated mice included animals that were positive for multiple criteria (Figure 2, C and D). Mice that met all 3 criteria represented 37% of morphine-treated mice and only 2% of saline-treated mice (Figure 2E). Mice that met all 3 positive criteria (D score = 3) were defined as depressive-like phenotype (Mor-D). Mice with a D score of 0 or 1 were defined as non-depressive-like phenotype (Mor-nD). The saline group only included saline-treated mice with a D score of 0 or 1. This model was used for subsequent experiments to accurately distinguish individual animals into subpopulations. In addition, we examined the correlation between each depressive-like behavior (Supplemental Table 3). We found significant correlations between sucrose preference and immobility times (Figure 2F), suggesting that depressive-like behaviors in TST, FST, and SPT were suitable for classifying depressive-like and non-depressive-like phenotypes in this mouse model.

Next, we asked whether Mor-D mice were vulnerable to stress-induced reinstatement to conditioned place preference (CPP). A new cohort of mice was trained with morphine to establish CPP (Figure 2, G and H). After confirmation of CPP extinction, mice were subjected to a subthreshold stress (foot shock) and immediately evaluated for CPP reinstatement. Interestingly, only Mor-D mice showed a significant reinstatement of CPP after foot shock (Figure 2I, left), while a priming dose of morphine (10 mg/kg, i.p.) elicited CPP reinstatement in both Mor-D and Mor-nD mice (Figure 2I, right). These results indicated that Mor-D mice were vulnerable to stress-induced reinstatement of CPP.

### Mice with depressive-like phenotypes show increased PVT→NAc^D2^ and decreased BLA→NAc^D1^ synaptic efficiency.

**Figure 3:**
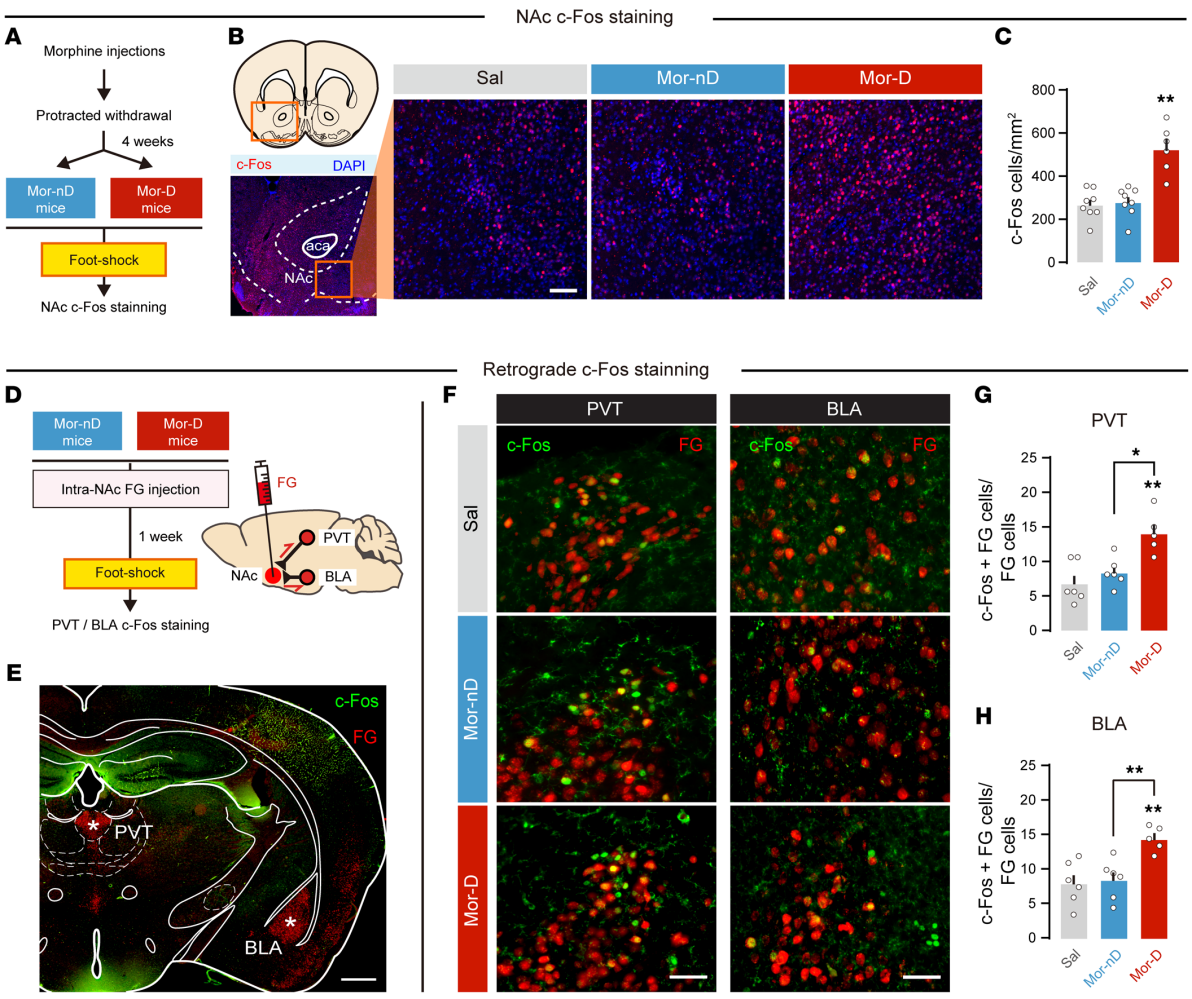
The BLA and PVT afferents to NAc are activated in mice with depressive-like behaviors after morphine withdrawal (Mor-D). (**A**) Flowchart of NAc c-Fos staining. (**B**) Representative NAc c-Fos immunostaining photos. Scale bar: 200 μm. (**C**) Total c-Fos–positive cells in NAc. Four slices per mouse, *n* = 6–8 per group. **P < 0.01, by 1-way ANOVA followed by Šidák’s test. (**D**) Flowchart of retrograde tracer Fluorogold (FG) injection and c-Fos staining in PVT or BLA. (**E**) Representative photo of c-Fos and FG staining in PVT and BLA. Scale bar: 0.5 mm. (**F**) Representative co-immunostaining photos for c-Fos and FG. Scale bars: 100 μm. (**G** and **H**) Active c-Fos cells (c-Fos+FG to total FG cells) in PVT and BLA. Four slices per mouse, *n* = 5–6 per group. *P < 0.05, **P < 0.01, by 1-way ANOVA followed by Šidák’s test. Data are presented as mean ± SEM.

The NAc is strongly implicated in opioid use disorder and depression (26, 27). We found robust c-Fos signals in NAc of Mor-D but not of Mor-nD or saline mice (Figure 3, A–C). To determine which afferents to NAc were activated in Mor-D mice, we injected retrograde tracer Fluorogold (FG) (Merck) into NAc and performed double labeling of c-Fos and FG in excitatory afferents to NAc (Figure 3, D and E, and Supplemental Figure 2, A and B). We observed an increase in active c-Fos cells (ratio of c-Fos+FG to total FG cells) in both PVT and BLA in Mor-D mice (Figure 3, F–H, and Supplemental Figure 2, C–E).

**Figure 4:**
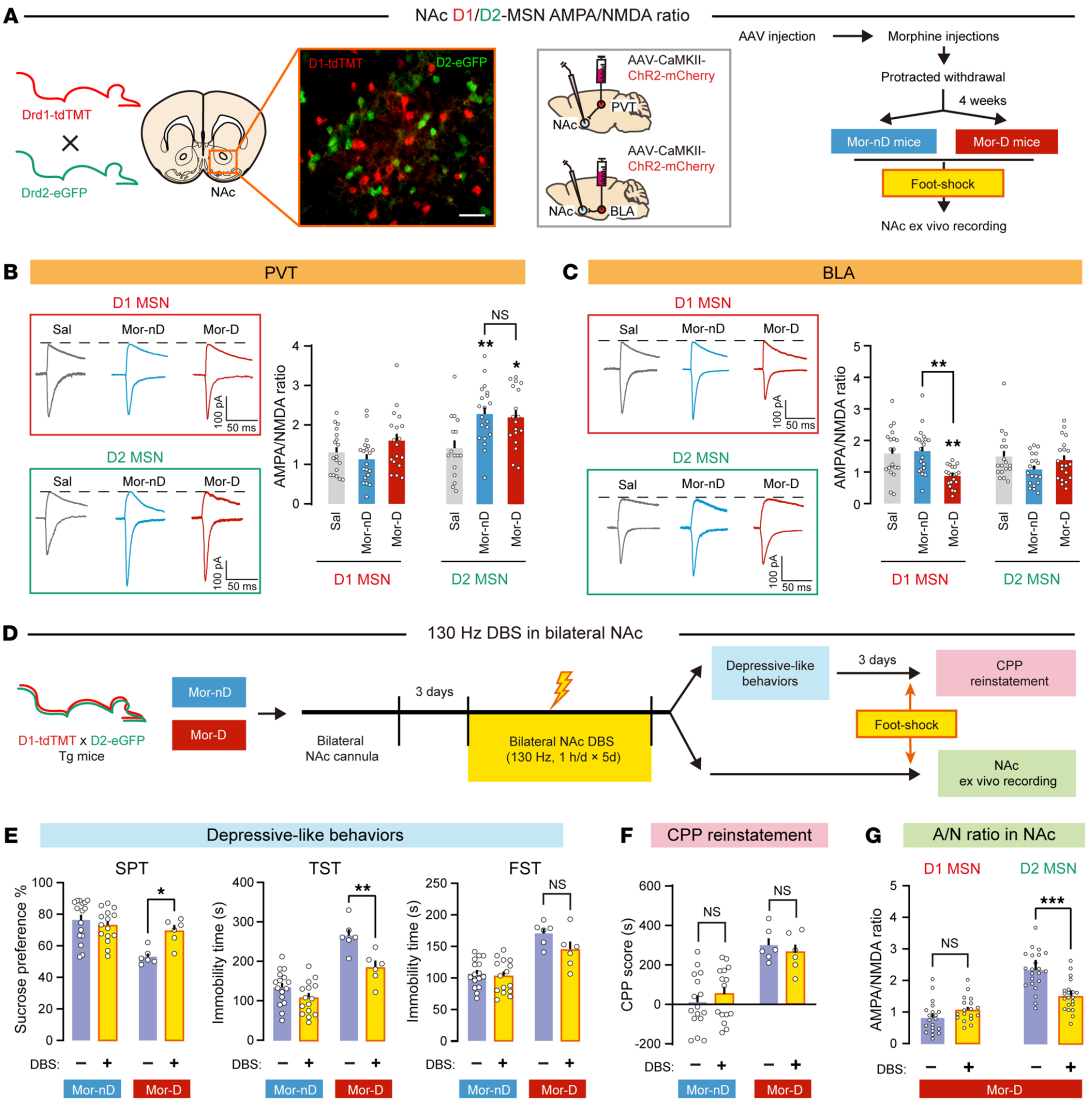
Mor-D mice show increased PVT→NAc^D2^ and decreased BLA→NAc^D1^ synaptic efficiency. (**A**) Schematic of the in vivo recording of NAc D1/D2 MSNs. D1-tdTomato×D2-eGFP double transgenic mice were used to identify NAc D1/D2 MSNs. Scale bar: 100 μm. (**B** and **C**) Sample of optically evoked EPSC traces of NAc D1 MSNs and D2 MSNs from PVT or BLA inputs. The A/N ratio is shown in NAc D1 MSNs and D2 MSNs. *n* = 16–20 neurons from 4–5 animals per group. *P < 0.05, **P < 0.01, by 1-way ANOVA followed by Tukey’s test. (**D**) Flowchart of the classical high-frequency DBS treatment in bilateral NAc (130 Hz, 1 h/d for 5 days). (**E**) Depressive-like behaviors in mice treated with or without 130 Hz DBS. *n* = 16 per group Mor-nD, and *n* = 6 per group Mor-D. *P < 0.05, **P < 0.01, by mixed RM 2-way ANOVA followed by Šidák’s test. (**F**) Foot shock–induced CPP reinstatement. (**G**) A/N ratio in NAc of Mor-D mice. *n* = 18–22 neurons from 4–6 animals per group. ***P < 0.0001, by unpaired Student’s *t* test. Data are presented as mean ± SEM.

To characterize synaptic efficiency in NAc D1- and D2-specific circuits, we injected ChR2-expressing virus (AAV-CaMK2-ChR2-mCherry) into PVT or BLA of D1-tdTomato×D2-eGFP double-transgenic mice. The whole-cell patch was performed ex vivo in identified NAc MSNs with optically evoked excitatory postsynaptic current (oEPSC) elicited by 473 nm light (Figure 4A). The ratio of AMPAR to NMDAR oEPSC (A/N ratio, which measures synaptic efficiency) increased in PVT→NAc^D2^ synapses of Mor-D and Mor-nD mice (Figure 4B). On the contrary, the A/N ratio was decreased only in BLA→NAc^D1^ synapses of Mor-D mice (Figure 4C), suggesting that BLA but not PVT afferents modulate depressive-like behaviors after morphine withdrawal. To test the effect of DBS treatment on depressive-like behaviors and stress-induced CPP reinstatement, we applied a classical high-frequency DBS (130 Hz, 90 ms, 1 h/d) to NAc shell for 5 days (Figure 4D). The DBS treatment only partially improved depressive-like behaviors in Mor-D mice (Figure 4E), but did not have any effect on foot shock–induced CPP reinstatement (Figure 4F). Ex vivo recording in NAc revealed that the A/N ratio was recovered only in D2 MSN synapses from DBS-treated Mor-D mice (Figure 4G). No changes were found in the A/N ratio in D1 MSN synapses.

### The PVT→NAc^D2^ pathway modulates aversive withdrawal symptoms in morphine -treated mice.

**Figure 5:**
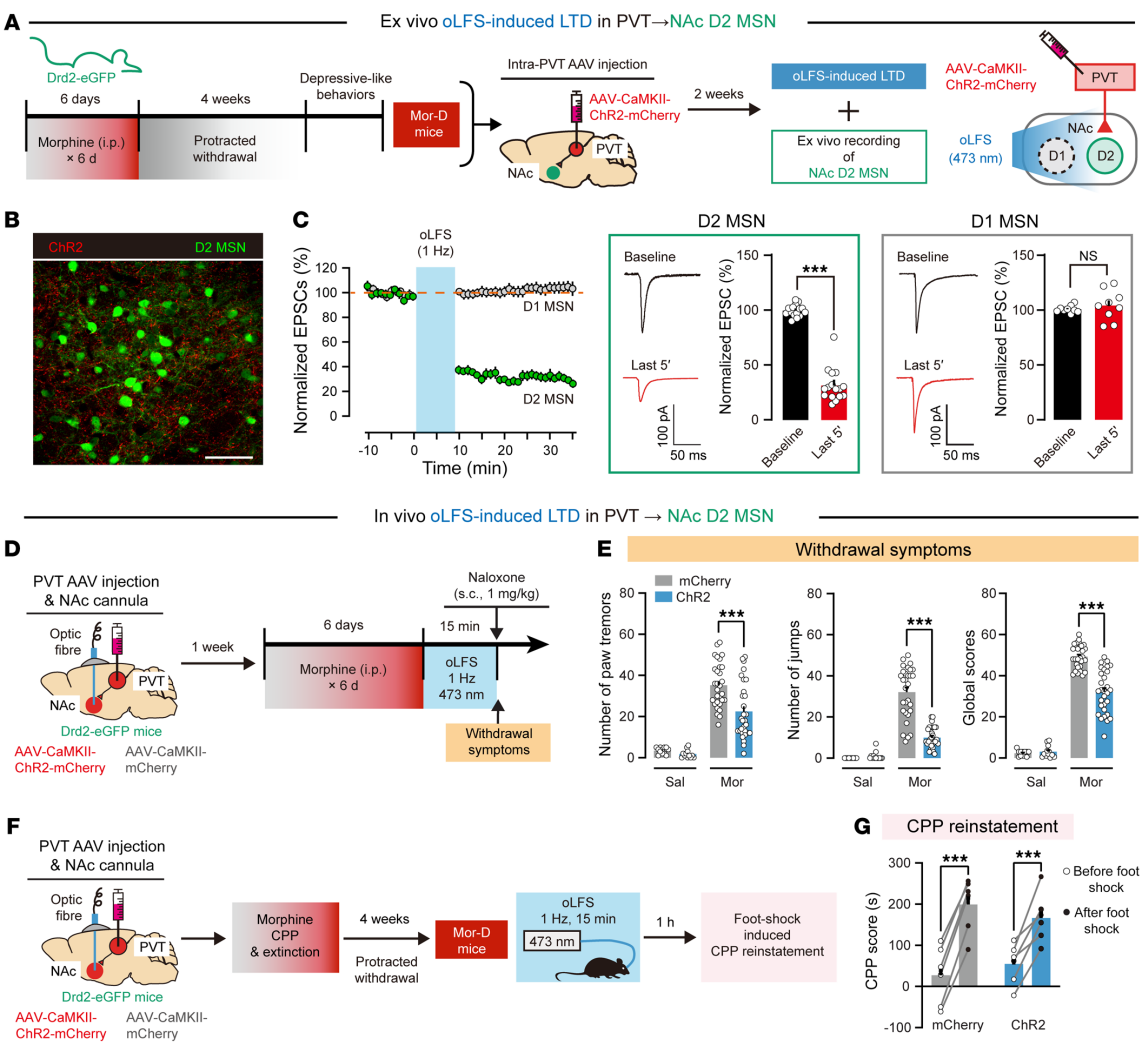
The PVT→NAc^D2^ pathway modulates aversive withdrawal symptoms in Mor mice. (**A**) Ex vivo oLFS-induced LTD in PVT→NAc D2 MSNs. The ChR2-expressing PVT→NAc pathway was stimulated by 1 Hz of oLFS to induce LTD. (**B**) Image of ChR2 expression in PVT terminals of D2-eGFP mice. Scale bar: 100 μm. (**C**) Normalized EPSC. oLFS induced a long-lasting reduction in EPSC in NAc D2 MSNs (*n* = 16 neurons from 4 mice) but not D1 MSNs (*n* = 9 neurons from 3 mice). ***P < 0.0001, by unpaired Student’s *t* test. (**D**) In vivo optogenetic depotentiation of PVT→NAc^D2^ pathway. Mice were implanted with optical fibers in dorsal to bilateral NAc and subjected to morphine treatment plus daily 1 Hz photo-stimulation for 15 minutes. (**E**) Naloxone-induced withdrawal symptoms in mice. *n* = 12–30 per group. ***P < 0.0001, by 2-way ANOVA followed by Šidák’s test. (**F**) Flowchart of CPP reinstatement test. Mor-D of D2-eGFP mice was subjected to daily 1 Hz photo-stimulations to induce oLFS in PVT→NAc^D2^ pathway. (**G**) Foot shock–induced CPP reinstatement. *n* = 6 per group. ***P < 0.0001, by mixed RM 2-way ANOVA followed by Šidák’s test. Data represent mean ± SEM.

Next, we investigated the role of the PVT→NAc^D2^ pathway in morphine withdrawal mice. Using D2-eGFP mice, we injected ChR2-expressing virus into PVT. In NAc slice, we applied ex vivo optogenetic low-frequency stimulation (oLFS; 1 Hz) to selectively induce long-term depression in PVT→NAc pathway (Figure 5, A and B). As previously described, D2 MSNs were visualized as eGFP-expressing neurons, while the MSNs that lacked the fluorescent tag were identified as D1 MSNs (10, 28, 29). oLFS induced a long-lasting EPSC reduction in D2 MSNs (31.5% ± 3.7% of baseline) and no obvious change in D1 MSNs (104.2% ± 4.62% of baseline) (Figure 5C). To test depotentiation of this pathway in vivo, D2-eGFP mice were injected with ChR2-expressing virus into PVT. After morphine administration, mice were subjected to 1 Hz oLFS treatment in NAc for 15 minutes (Figure 5D). oLFS significantly reduced the level of withdrawal symptoms induced by naloxone in the ChR2 group compared with the control group (Figure 5E). Furthermore, 4 weeks after withdrawal, ChR2 mice treated with oLFS showed similar depressive-like behaviors and depression ratio to control mice (23.4% vs. 26.6%, *n* = 30 per group) (Supplemental Figure 3, A–C). We also found that Mor-D mice treated with oLFS (Figure 5F) showed a similar stress-induced reinstatement of CPP in both ChR2 and control groups (Figure 5G). These results demonstrated that the aversive withdrawal symptoms were encoded by the PVT→NAc^D2^ pathway. Importantly, the circuits that encode withdrawal symptoms and depressive components may differ in the brain.

### KOR on BLA→NAc pathway is essential for depressive-like behaviors in Mor-D mice.

**Figure 6:**
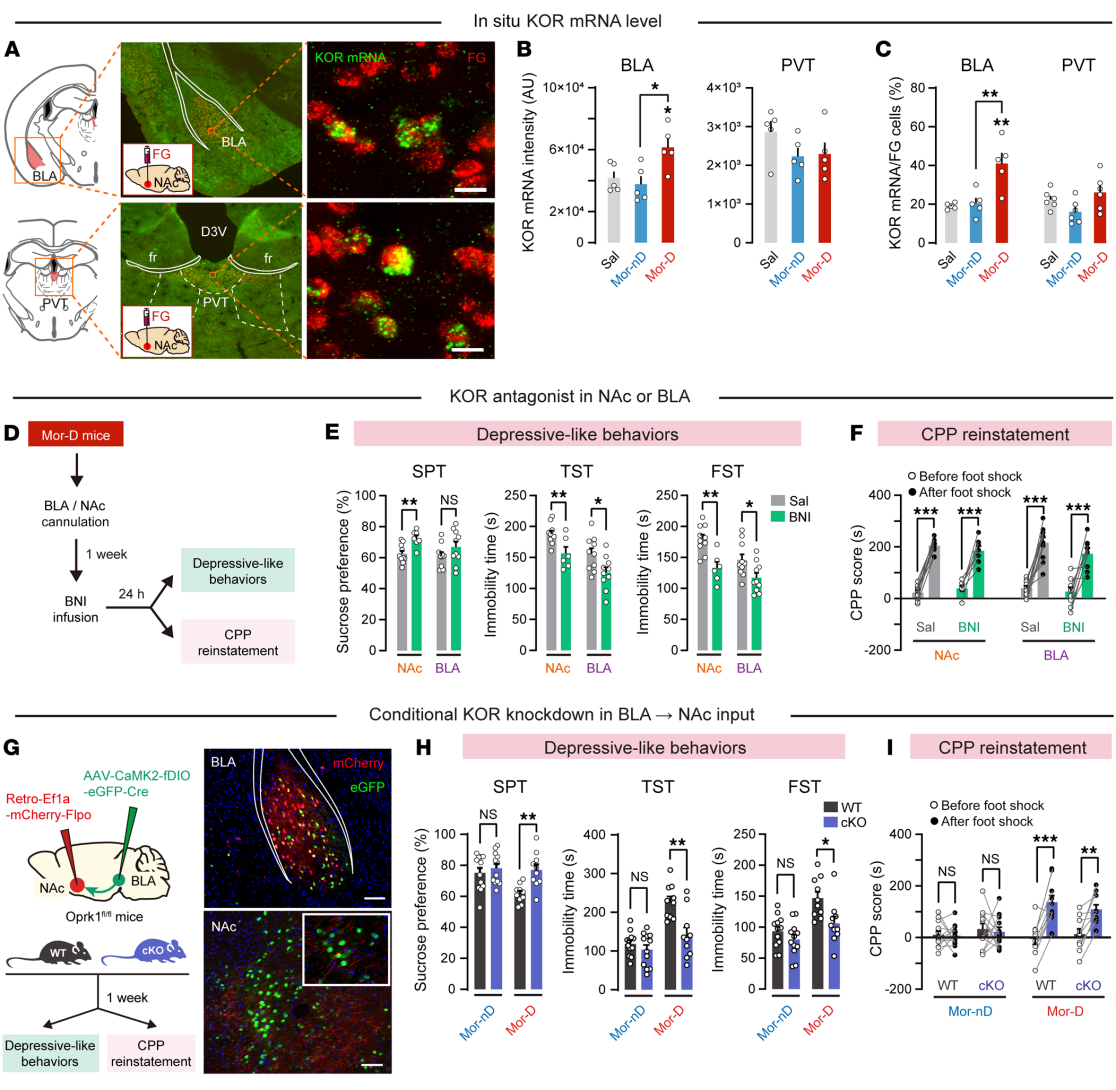
The KOR on BLA→NAc pathway is essential for Mor-D mice. (**A**) Photos of in situ hybridization of KOR mRNA and Fluorogold (FG) in BLA and PVT. fr, fasciculus retroflexus. Scale bars: 50 μm. (**B**) Mean KOR mRNA intensity in BLA and PVT. *n* = 5 per group. *P < 0.05, by 1-way ANOVA followed by Tukey’s test. (**C**) Percentage of KOR mRNA plus FG double-labeled cells in BLA and PVT. *n* = 5–6 per group. **P < 0.01, by 1-way ANOVA followed by Tukey’s test. (**D**) Flowchart of behavioral tests after norBNI infusion. The KOR antagonist norBNI (5 μg/μL, 0.5 μL/side) was infused bilaterally into NAc or BLA of Mor-D mice 24 hours before behavioral tests. (**E**) Depressive-like behaviors in norBNI-treated mice. *n* = 7–10 per group. *P < 0.05, **P < 0.01, by unpaired Student’s *t* test. (**F**) Foot shock–induced CPP reinstatement. *n* = 8–10 per group. ***P < 0.0001, by mixed RM 2-way ANOVA followed by Šidák’s test. (**G**) Conditional KOR knockdown on BLA→NAc pathway. Scale bars: 100 μm. (**H**) Depressive-like behaviors in the wild-type (WT) or conditional-KOR-knockdown (cKO) mice. *n* = 10–12 per group. *P < 0.05, **P < 0.01, by unpaired Student’s *t* test. (**I**) Foot shock–induced CPP reinstatement in the WT or cKO mice. *n* = 9 per group. **P < 0.01, ***P < 0.0001, by mixed RM 2-way ANOVA followed by Šidák’s test. Data are presented as mean ± SEM.

We noticed that KOR dynamically shapes the NAc D1 and D2 MSN collaterals (10). In NAc, we found that the μ-opioid receptor (MOR) was elevated in Mor-D and Mor-nD mice, while the levels of KOR and prodynorphin in NAc were elevated only in Mor-D mice (Supplemental Figure 4, A–D). This result indicates that the abnormal increase in NAc KOR and dynorphin levels may correspond to a greater risk of developing depressive-like behaviors in morphine withdrawal mice. To determine whether differential modulation of PVT and BLA afferents was due to differential expression of KOR, we injected retrograde tracer FG into NAc and performed KOR mRNA in situ hybridization in PVT and BLA (Figure 6A). In BLA but not PVT, we observed an increase in the number of double-labeled cells with FG and KOR mRNA in Mor-D mice (Figure 6, B and C), indicating that KOR expression was increased in BLA→NAc inputs. To detect whether KOR in NAc regulates depressive-like behavior in Mor-D mice, we bilaterally infused a long-acting KOR antagonist, norbinaltorphimine (norBNI; 5 μg/μL, 0.5 μL per side), into BLA or NAc 24 hours before the behavioral test (Figure 6D). We found that depressive-like behaviors in Mor-D mice were ameliorated by KOR blockade (Figure 6E). However, the CPP scores in the 2 norBNI groups were similar to those of the saline controls, indicating that KOR blockade in NAc or BLA failed to prevent the CPP reinstatement in these mice (Figure 6F).

To further investigate the specific role of presynaptic KOR in NAc, we used transgenic KOR-floxed mice (Oprk1^fl/fl^) combined with the virus-mediated FLP/FRT system to conditionally eliminate KOR on BLA→NAc inputs. As we previously described (30), the Oprk1^fl/fl^ mice were stereotaxically injected with AAV-CaMK2-fDIO-eGFP-2A-Cre into BLA and Retro-AAV-Ef1a-mCherry-Flpo into NAc for Cre recombinase expression (Figure 6G). Interestingly, the elimination of KOR on BLA→NAc pathway significantly ameliorated depressive-like behaviors in Mor-D mice (Figure 6H), but did not prevent the reinstatement of CPP in these mice (Figure 6I). Taken together, these results revealed that the KOR on BLA→NAc pathway is essential for depressive-like behaviors in Mor-D mice.

### The BLA→NAc^D1^ pathway bidirectionally modulates depressive-like behavior and stress-induced CPP reinstatement.

**Figure 7:**
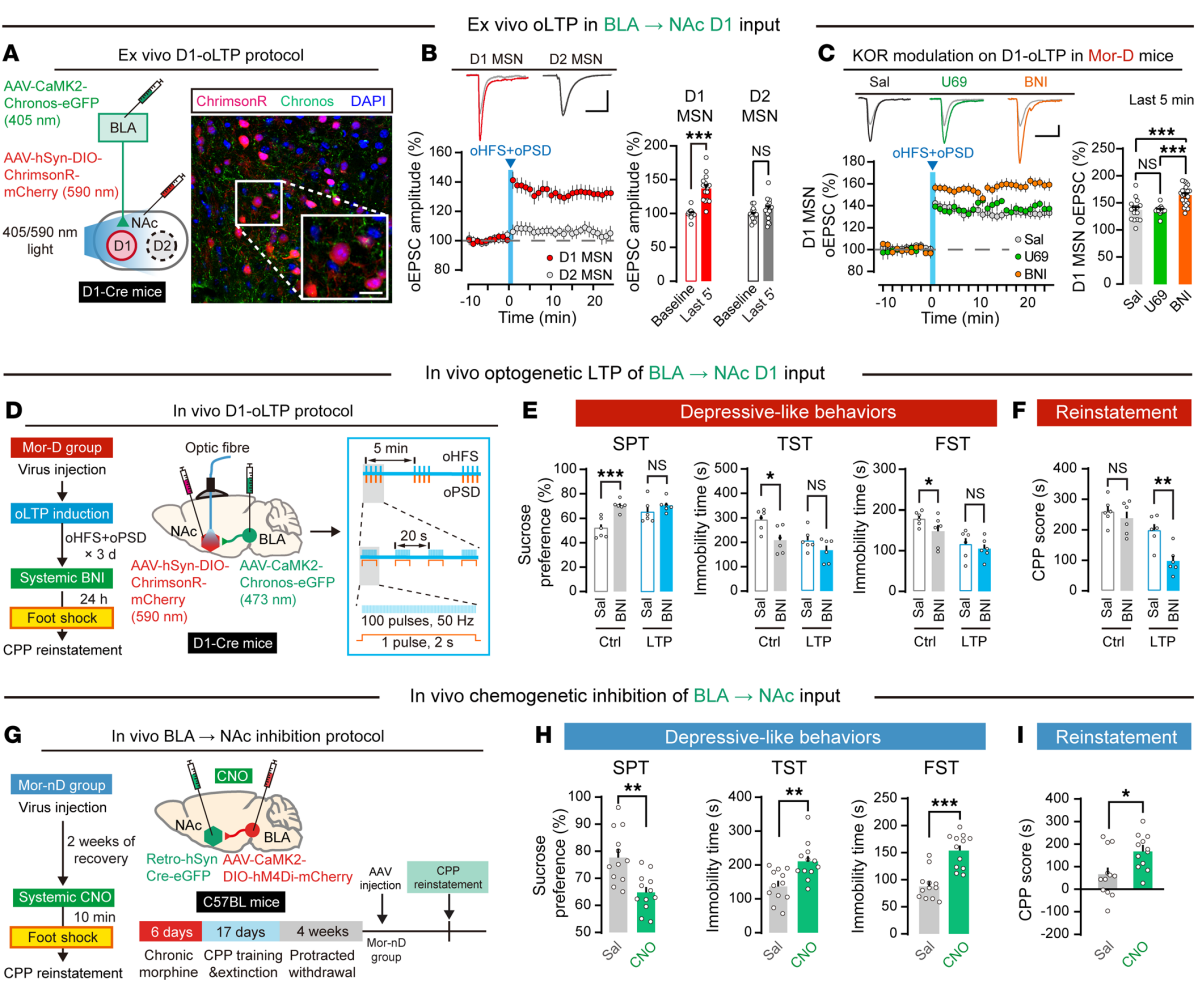
BLA→NAc^D1^ pathway bidirectionally modulates depressive-like behavior and stress-induced CPP reinstatement. (**A**) Dual-channel ex vivo optogenetic LTP protocol in D1-Cre mice. Chronos and ChrimsonR were activated by 405 nm and 590 nm light, respectively. Scale bar: 100 μm. (**B**) Optogenetic high-frequency stimulation (oHFS; 405 nm, 50 Hz, 100 pulses, repeated 4 times with a 20-second interval) combined with optogenetic postsynaptic depression (oPSD; 2 seconds, 590 nm) induced a long-lasting elevation of EPSC in BLA→NAc^D1^ pathway. *n* = 15 neurons from 3 animals. ***P < 0.0001, by unpaired Student’s *t* test. Scale bars: 50 ms and 50 pA. (**C**) oHFS + oPSD + U69 or oHFS + oPSD + norBNI induced a long-lasting increase in EPSC in BLA→NAc^D1^ pathway. *n* = 15–22 neurons in 3–6 animals per group. ***P < 0.0001, by 1-way ANOVA followed by Tukey’s test. (**D**) Flowchart of the dual-channel in vivo optogenetic LTP protocol. Chronos and ChrimsonR were expressed in BLA→NAc^D1^ afferents of Mor-D mice. (**E**) Depressive-like behaviors in D1-oLTP treatment. *n* = 6 per group. *P < 0.05, ***P < 0.0001, by 2-way ANOVA followed by Šidák’s test. (**F**) Foot shock–induced CPP reinstatement. *n* = 6 per group. **P < 0.01, by 2-way ANOVA followed by Šidák’s test. (**G**) Schematic of the in vivo chemogenetic inhibition protocol. hM4Di was expressed in BLA→NAc afferents of Mor-nD mice. CNO was administered before foot shock. (**H**) Depressive-like behaviors after CNO administration. *n* = 12 per group. **P < 0.01, ***P < 0.0001, by unpaired Student’s *t* test. (**I**) Foot shock–induced CPP reinstatement. *n* = 12 per group. *P < 0.05, by unpaired Student’s *t* test. Data are presented as mean ± SEM.

Next, we explored the role of BLA→NAc^D1^ pathway in Mor-D mice. To selectively induce long-term potentiation (LTP) in BLA→NAc^D1^ pathway, we used an ex vivo dual-channel optogenetic LTP protocol as previously reported (31). As shown in Figure 7A, D1 MSNs were identified by expression of mCherry in D1-Cre mice (injection of AAV-hSyn-DIO-ChrimsonR-mCherry into NAc), which were specifically activated by 590 nm wavelength. D2 MSNs did not show a fluorescent tag or a synaptic response to 590 nm optogenetic stimulation, while like the D1 MSNs, they showed a response to 405 nm stimulation (injection of AAV-CaMK2-Chronos-eGFP into BLA) as D1 MSNs (10, 28, 29). In NAc slices, we found that Chronos-mediated optogenetic high-frequency stimulation (oHFS) with ChrimsonR-mediated optogenetic postsynaptic depolarization (oPSD) induced robust LTP in D1 but not D2 MSNs from Mor-D mice (Figure 7B). In contrast, LTP was not observed in D1 MSNs when only oHFS or oPSD was applied (Supplemental Figure 5A). To investigate whether KOR affects LTP of BLA→NAc^D1^ pathway in Mor-D mice, we pretreated NAc slices with the KOR agonist U69,593 (1 μM, 10 minutes before LTP) or the antagonist norBNI (10 μM, 1 hour before LTP) before the dual-channel optogenetic long-term potentiation (oLTP) protocol in D1 MSNs (10, 32). Compared with Mor-nD mice and saline controls, we observed a greater oEPSC in D1 MSNs in Mor-D mice from both the U69 group (oEPSC amplitude 136.8%; Supplemental Figure 5B) and the norBNI group (oEPSC amplitude 164.3%; Supplemental Figure 5C). Furthermore, oEPSC in D1 MSNs of Mor-D mice was significantly enhanced by pretreatment with norBNI but not U69 (Figure 7C and Supplemental Figure 5D), suggesting that KOR blockage selectively amplified the potentiation of BLA→NAc^D1^ pathway in Mor-D mice.

Using D1-Cre mice, we applied an in vivo oLTP protocol to potentiate BLA→NAc^D1^ pathway. Chronos and ChrimsonR were specifically expressed in BLA→NAc^D1^ pathway in Mor-D mice. Dual-channel light stimulation was delivered to NAc for 3 days. Meanwhile, norBNI (20 mg/kg, s.c.) was administered 24 hours before behavioral tests (33) (Figure 7D). We found that this in vivo LTP with norBNI treatment significantly improved depressive-like behaviors in Mor-D mice (Figure 7E). Furthermore, this treatment effectively abolished stress-induced CPP reinstatement (Figure 7F).

To further confirm the necessity of the BLA→NAc pathway for depressive-like behaviors, we selectively inhibited BLA→NAc pathway in Mor-nD mice with a chemogenetic approach (Figure 7G). We found that inhibition of BLA→NAc pathway significantly decreased sucrose preference and increased immobility time in Mor-nD mice (Figure 7H). Furthermore, Mor-nD mice with inhibition of BLA→NAc pathway showed significant stress-induced CPP reinstatement, a phenomenon similar to that observed in Mor-D mice (Figure 7I). These results clearly indicated that activation of BLA→NAc^D1^ pathway is necessary for homeostatic resilience to depressive-like behaviors and stress-induced CPP reinstatement.

### Refined DBS treatment improves depressive-like behaviors and inhibits stress-induced CPP reinstatement in Mor-D mice.

**Figure 8:**
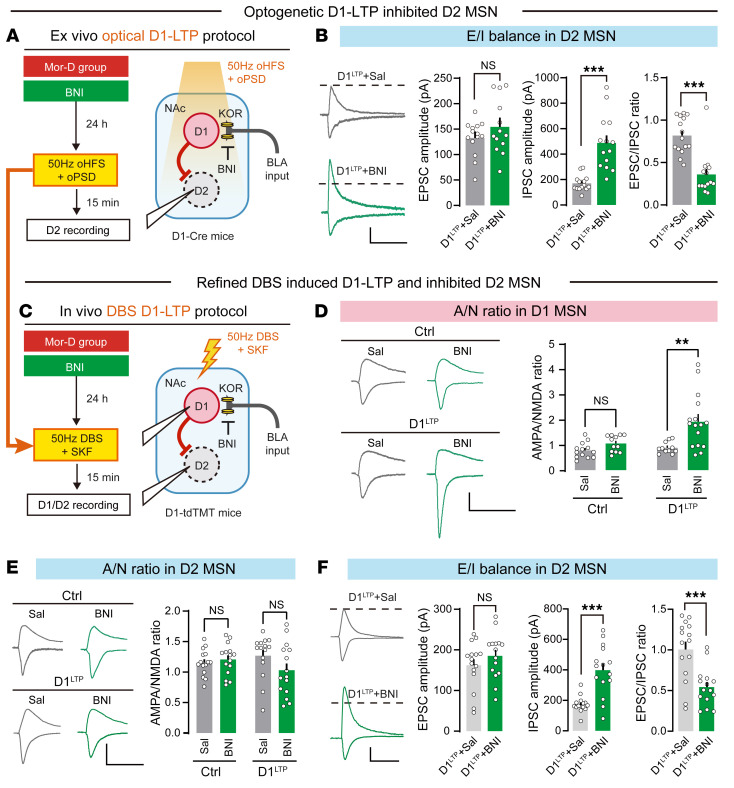
Refined DBS increases the A/N ratio in NAc D1 MSNs. (**A**) Schematic for excitation/inhibition (E/I) balance of NAc D2 MSNs. After induction of oLTP in BLA→NAc D1 MSNs with or without norBNI treatment, presumptive NAc D2 MSNs (MSNs without fluorescence) were recorded for EPSC and IPSC. (**B**) The LTP+norBNI group showed a lower E/I ratio. *n* = 14–15 neurons in 5 animals per treatment group. ***P < 0.0001, by unpaired Student’s *t* test. Scale bar: 50 ms, 50 pA. (**C**) Schematic of the in vivo DBS-induced LTP protocol in Mor-D mice. The refined in vivo DBS protocol includes 50 Hz DBS, D1 agonist (SKF38393), and norBNI treatment. NAc D1 MSNs (MSNs with red fluorescence) were recorded for EPSC of AMPAR and NMDAR. (**D** and **E**) The A/N ratio in NAc D1 MSNs (**D**) and D2 MSNs (**E**). Left: Example EPSC traces. Right: A/N ratio of NAc D1 MSNs. Scale bars: 50 ms, 100 pA. (**F**) The E/I balance in D2 MSNs. Left: Example IPSC traces. Right: E/I ratio of NAc D2 MSNs. *n* = 12–15 neurons in 5 animals per treatment group. Scale bar: 50 ms, 50 pA. **P < 0.01, ***P < 0.0001, by unpaired Student’s *t* test. Data are presented as mean ± SEM.

Recent evidence suggested that KOR inhibits D1 MSN collaterals more strongly than it inhibits D2 MSN collaterals (10). We hypothesized that enhancement of NAc D1 MSNs with presynaptic KOR antagonism in the BLA neuron alters the excitation/inhibition (E/I) balance of NAc D2 MSNs. Using the ex vivo D1 MSN oLTP protocol, we measured the optogenetic evoked E/I ratio in NAc D2 MSNs from previously used Mor-D mice (Figure 8A). The E/I ratio of D2 MSNs (without fluorescence tag) was reduced in the D1^LTP^+norBNI group compared with the D1^LTP^+Sal group (Figure 8B), suggesting that KOR on BLA terminals selectively regulates collateral inhibition of D1 MSNs to D2 MSNs. Next, we sought to translate optogenetic D1-LTP protocol to refined in vivo DBS treatment. Using the D1-tdTomato mice, we performed NAc DBS at 50 Hz (translated from oHFS frequency) to determine whether BLA→NAc^D1^ pathway is enhanced in Mor-D mice (Figure 8C). D1 receptor agonism (SKF38393, 1.40 mg/kg, i.p.) was performed simultaneously to mimic optogenetically induced oPSD. Previous studies have shown that excitability in D1 MSNs increases after administration of the D1 agonist (34–36). We found that only KOR blockade (norBNI, 20 mg/kg, s.c.) (37) with DBS+SKF, but not any single treatment itself, selectively elevated A/N ratios in NAc D1 but not D2 MSNs (Figure 8, D and E, and Supplemental Figure 6, A and B). Furthermore, the E/I ratio of D2 MSNs was reduced in the norBNI group (Figure 8F). These results suggested that the combined approach of 50 Hz DBS plus D1 receptor agonism with KOR blockade effectively potentiated NAc D1 MSNs and amplified the collateral inhibition of D2 MSNs, reducing the D2 receptor–mediated influence.

**Figure 9:**
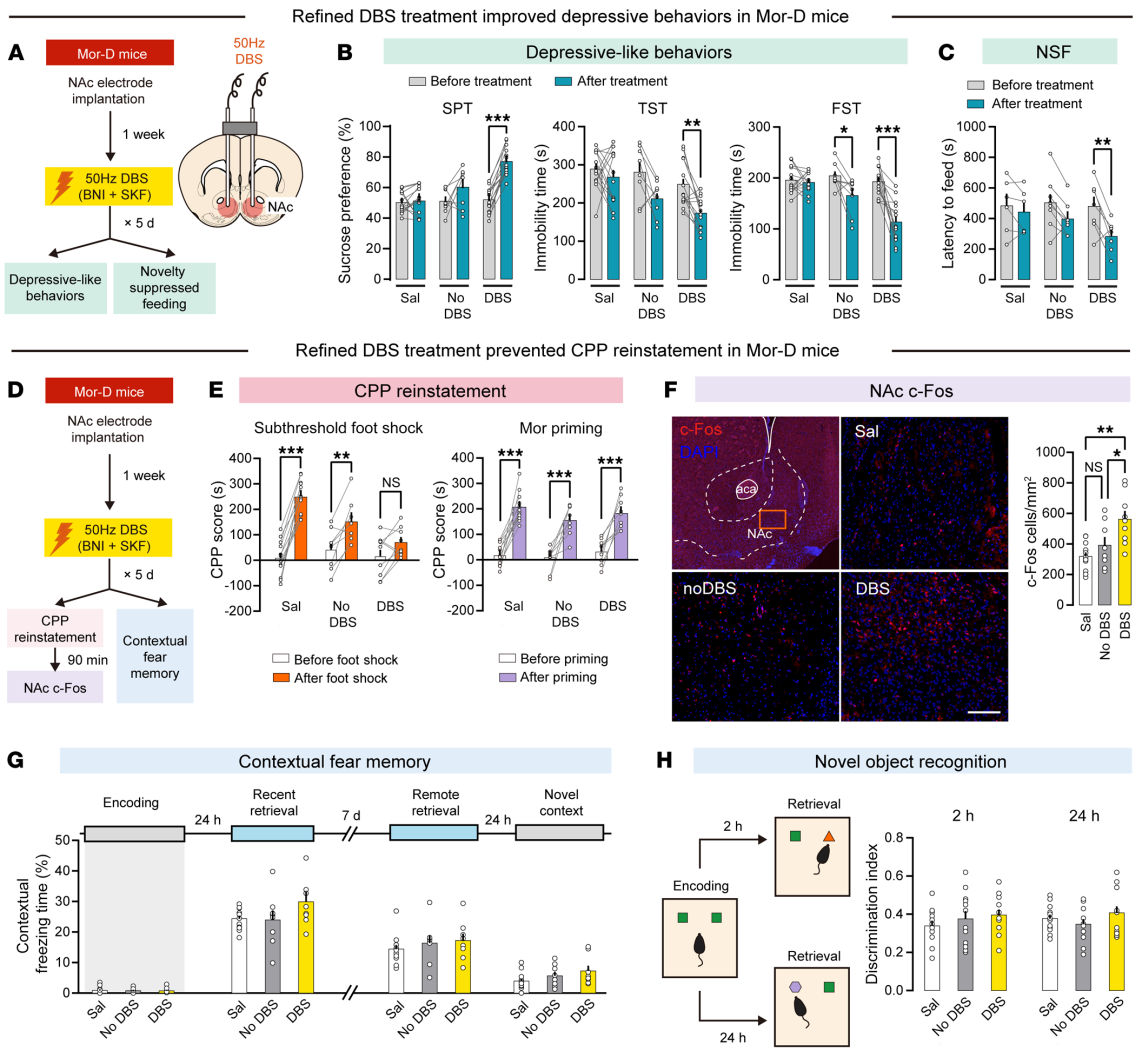
Refined DBS treatment improves depressive-like behaviors and inhibits stress-induced CPP reinstatement in Mor-D mice. (**A**) Flowchart and schematic of the refined DBS treatment. Mor-D mice were implanted with bilateral NAc electrodes and treated with norBNI + SKF + DBS for 5 days. (**B**) Depressive-like behaviors before and after refined DBS treatment. *n* = 12–13 per group. *P < 0.05, **P < 0.01, ***P < 0.0001, by mixed 2-way ANOVA followed by Šidák’s test. (**C**) Novelty-suppressed feeding (NSF) test. *n* = 6–8 per group. **P < 0.01, by mixed 2-way ANOVA followed by Šidák’s test. (**D**) Flowchart of CPP reinstatement and memory tests. (**E**) Foot shock– or morphine-induced CPP reinstatement. *n* = 7–12 per group. **P < 0.01, ***P < 0.0001, by mixed 2-way ANOVA followed by Šidák’s test. (**F**) Immunostaining of NAc c-Fos following the CPP reinstatement test. Scale bar: 200 μm. Four slices per mouse, *n* = 6–8 per group. *P < 0.05, **P < 0.01, by mixed 1-way ANOVA followed by Tukey’s test. (**G**) Contextual freezing time in different memory stages. *n* = 10–12 per group. Data were analyzed by 1-way ANOVA. (**H**) Discrimination index in different memory stages. Left: Schematic for the novel object recognition test. Right: Discrimination index. *n* = 10–12 per group. Data were analyzed by 1-way ANOVA. Data are presented as mean ± SEM.

We further tested this refined DBS treatment at the system level (Figure 9A). This treatment significantly improved depressive-like behaviors in Mor-D mice (Figure 9B). The validity of this treatment was also confirmed by the novelty-suppressed feeding (NSF) test. Refined DBS–treated mice showed significantly lower latency to feed than other groups, suggesting improved anhedonia in these mice when in a novel open space (Figure 9C). We then tested the effect of refined DBS treatment on CPP reinstatement and memory retrieval in Mor-D mice (Figure 9D). Interestingly, refined DBS treatment fully prevented stress-induced reinstatement of CPP in Mor-D mice (Figure 9E, left). Additionally, a priming dose of morphine (10 mg/kg, i.p.) still elicited a strong reinstatement of CPP (Figure 9E, right), suggesting an intact memory retrieval of morphine CPP. Analysis of c-Fos staining revealed a significant increase in NAc c-Fos cells in DBS-treated mice (Figure 9F), suggesting that NAc cells were activated in these mice. We also performed a state-dependent contextual fear memory test and a novel object recognition test to further evaluate memory encoding and retrieval in DBS-treated mice. As shown in Figure 9G, DBS-treated mice showed normal encoding and recent (24 hours) and remote (7 days) retrieval of fear memory compared with control mice. Furthermore, the discrimination index in the novel object recognition test was similar between 3 groups (Figure 9H). Together, these results suggested unchanged memory formation and recall after the refined DBS treatment.

### Refined DBS treatment prevents naloxone-induced withdrawal symptoms and improves depressive-like behaviors and stress-induced CPP reinstatement in both male and female mice.

**Figure 10:**
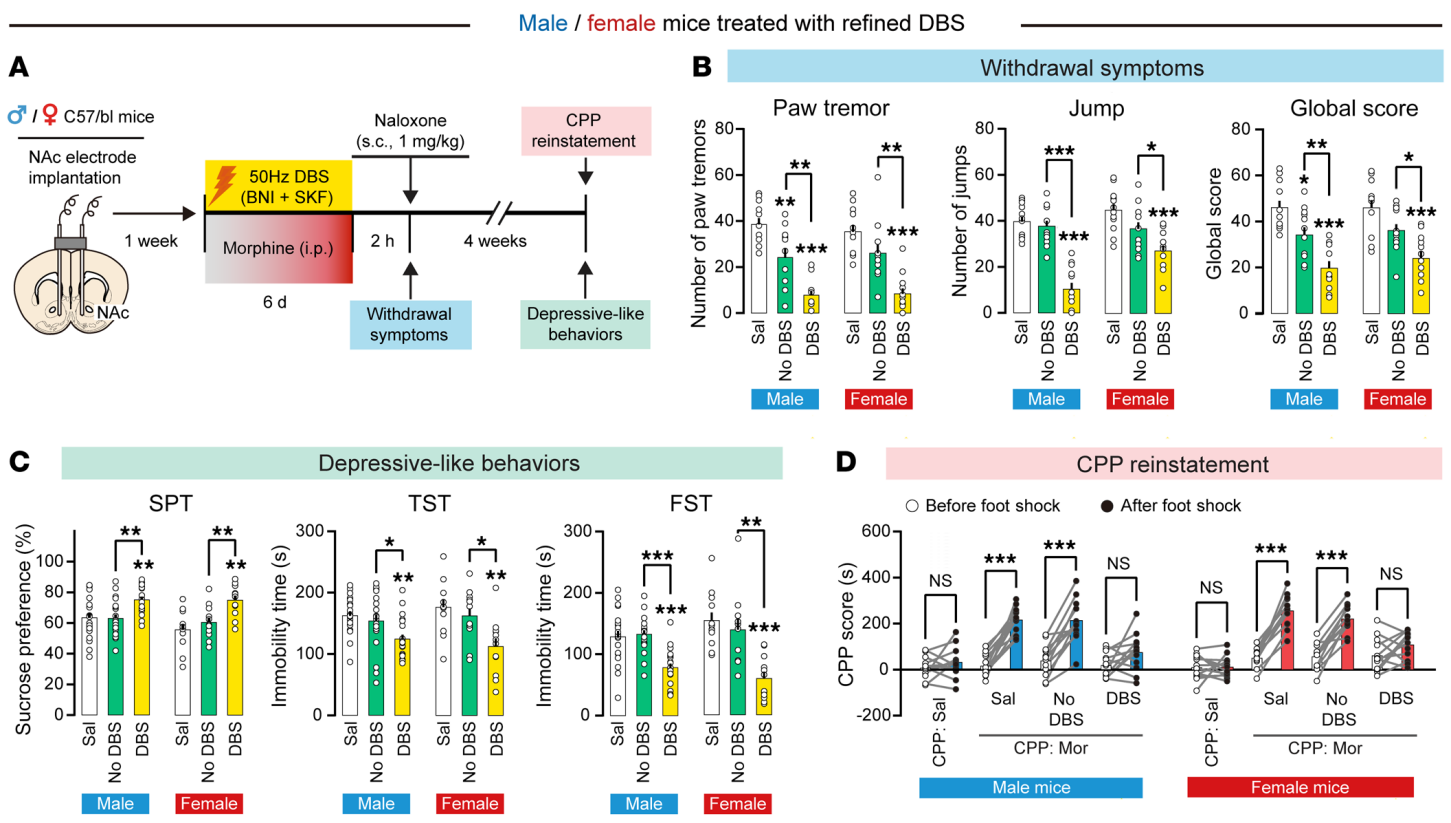
Refined DBS treatment in male and female mice. (**A**) Schematic and flowchart of refined DBS treatment in male and female mice. (**B**) Naloxone-induced withdrawal symptoms in male and female mice following refined DBS treatment. *n* = 10 per group. *P < 0.05, **P < 0.01, ***P < 0.0001, by 1-way ANOVA followed by Tukey’s test. (**C**) Depressive-like behaviors in male and female mice following refined DBS treatment. *n* = 14–17 per group. *P < 0.05, **P < 0.01, ***P < 0.0001, by 1-way ANOVA followed by Tukey’s test. (**D**) Foot shock–induced CPP reinstatement in male and female mice following the refined DBS treatment. *n* = 12–17 per group. ***P < 0.0001, by mixed RM 2-way ANOVA followed by Šidák’s test. Data are presented as mean ± SEM.

We further proposed that both naloxone-induced withdrawal symptoms (controlled by PVT→NAc^D2^ pathway) and depressive-like behaviors (controlled by BLA→NAc^D1^ pathway) could be improved, resulting in resilience to stress-induced CPP reinstatement. Refined DBS treatment (1 h/d for 6 days) was applied to morphine-treated male and female mice 24 hours before behavioral tests (Figure 10A). The refined DBS treatment significantly improved naloxone-induced withdrawal symptoms in both male and female mice (Figure 10B). Furthermore, this treatment also significantly alleviated depressive-like behaviors in male and female mice (Figure 10C and Supplemental Figure 7A). Importantly, this refined DBS treatment effectively prevented stress-induced CPP reinstatement in both male and female mice (Figure 10D and Supplemental Figure 7, B–D). These results indicate that our DBS treatment with KOR antagonism is effective in the treatment of morphine withdrawal–related behaviors in both male and female mice.

## Discussion

Our results revealed synaptic causalities underlying withdrawal symptoms and depressive-like behaviors following morphine withdrawal, which are common but understudied in the context of drug use disorder. Our behavioral screening method enabled us to distinguish a subpopulation of morphine-treated mice with obvious depressive-like behaviors; these mice were prone to stress-induced reinstatement. Using this approach, we found that excitatory afferents arising from PVT and BLA differentially control D2 and D1 MSNs in NAc, leading to somatic and emotional abnormalities after withdrawal. Moreover, we found that optogenetically induced LTP of the BLA→NAc^D1^ circuit with KOR blockade efficiently prevented depressive-like behaviors and stress-induced restatement by rebalancing the local circuits of NAc D1 and D2 MSNs. Importantly, these results could be replicated using a refined DBS protocol.

First, we established a reliable behavioral paradigm to screen mice with significant depressive-like behaviors after withdrawal. Opioid use disorder patients with depression have longer detoxification times, worse prognoses, and a higher relapse rate. However, the effect of antidepressants on comorbid depression in patients with drug use disorder is generally limited or negative (38, 39). Until now, no single animal model fully represented these depressive behaviors (40). Furthermore, few investigators have explicitly examined heterogeneity of emotional alterations in morphine withdrawal animals (3). To our knowledge, our results show for the first time that depression vulnerability differentially affects stress-induced reinstatement (41). In present study, we directly retrieved metrics from 3 well-established paradigms of depressive behaviors of morphine withdrawal mice. We found that 37% of morphine withdrawal mice met all 3 criteria. Thus, we classified morphine withdrawal mice into depressive (Mor-D) and non-depressive (Mor-nD) groups.

Second, we found that KOR regulates the excitation/inhibition balance of NAc D1 MSNs to D2 MSNs through lateral inhibition. Consistent with the notion that KOR activation in animals and humans produces negative emotional states and drug-seeking behaviors (16, 42, 43), we found elevated expression of KOR in NAc of Mor-D mice, leading to enhanced inhibitory drive from D1 MSNs to D2 MSNs (since the action of KOR within NAc is D1 dominant) (10). Furthermore, we found that a high level of KOR mRNA originates from the BLA projection in Mor-D mice. Pretreatment with the KOR agonist U69 did not affect the oLTP of D1 MSNs in both groups. However, we observed that the KOR antagonist norBNI amplified the oLTP of D1 MSNs exclusively in Mor-D group. These results highlight the crucial role of KOR in modulating the behavioral state–dependent effect in a projection pathway–specific manner.

BLA to NAc afferents are thought to encode both positive and negative emotional valence (14, 44). Intriguingly, no change in BLA-projecting cell type–specific plasticity was found in cocaine seeking (13) and opioid withdrawal (8). We found significant synaptic depression of BLA→NAc^D1^ pathway only in Mor-D mice. Chemogenetically induced inhibition of BLA→NAc^D1^ pathway in Mor-nD mice reinstated depressive behaviors and stress-induced CPP. Optogenetic potentiation of BLA→NAc^D1^ pathway in Mor-D mice via an in vivo LTP protocol prevented depressive phenotypes. We also found that depotentiating PVT→NAc^D2^ pathway in vivo restored its normal transmission and relieved withdrawal symptoms. Thus, we offer 2 important findings regarding depressive-like states after opioid withdrawal (8). First, the negative emotional component is encoded in the BLA→NAc^D1^ circuit, which could trigger reinstatement of drug-seeking behavior through exposure to stress. Second, the negative somatic state that occurs after morphine withdrawal is regulated by plasticity in the PVT→NAc^D2^ circuit.

DBS represents a promising therapeutic option for drug use disorder and refractory depression (45–48). Drug seeking involves complex changes in synaptic plasticity in an input- and cell type–specific manner (8, 13); thus, a refined DBS protocol aimed at addressing synaptic pathology represents a potential new approach to treatment of drug use disorder (23). Our study investigates the potential of DBS as a clinical approach to address circuit dysfunction associated with depressive-like behaviors induced by morphine withdrawal. To achieve this, we used a combined approach of optogenetics and genetic-targeted tools in mice to manipulate intermixed NAc populations. We found that these interventions improved circuit dysfunction and helped alleviate depressive-like behaviors. We refined our approach by developing 50 Hz DBS protocols that replicated our optogenetic manipulations. The refined DBS approach involved exciting D1-expressing neurons through the use of D1 agonist, while also blocking KOR to amplify collateral inhibitory input from D1 MSNs to D2 MSNs. This population-specific neuromodulation was achieved with our DBS protocol and resulted in outcomes similar to those seen with our optogenetic approach.

One major adjustment in our DBS protocol was inclusion of KOR antagonist, which may have benefits for treatment of mood disorders and drug use disorder by promoting resilience to stress (49), but has little effect on opioid reinstatement when administered alone. Currently, high-frequency (>100 Hz) DBS is unable to restore normal synaptic transmission in NAc (23). Based on optogenetically induced LTP protocol in NAc D1 signaling, we showed that 50 Hz DBS of NAc combined with KOR antagonism and D1 receptor activation effectively ameliorated depressive phenotypes, and prevented stress-induced morphine reinstatement. Another interesting finding of our study is that increased excitatory transmission in NAc D2 signaling in Mor-D mice could be counterbalanced by elevation of local inhibitory transmission caused by potentiation of D1 signaling. Reversal of synaptic depression in BLA→NAc^D1^ circuit through KOR antagonism could reduce activity of NAc D2 MSNs by disinhibiting D1 MSNs to D2 MSNs lateral. Our refined DBS treatment not only improved emotional abnormalities via potentiation of BLA→NAc^D1^ pathway, but also alleviated withdrawal symptoms by restoring excitation and inhibition balance of NAc D2 MSNs.

We acknowledge several limitations of this study. As demonstrated in Figure 2, we found a high variability in depressive-like behaviors in mice after protracted morphine withdrawal, which enables us to distinguish subgroups of depressive and non-depressive phenotypes sufficiently. It is a very interesting phenomenon, as these mice share identical genetic backgrounds and received treatment in the same order. However, we did not analyze any correlation between behavioral phenotypes (Mor-D and Mor-nD) and housing conditions or treatment order. Regarding empathetic transfer/expectancy, we did not observe any factors that influenced the phenotype, since the Mor-D and Mor-nD ratio among the groups was similar, and random treatments were administered. Social dominance was not assessed in this study; therefore, we cannot determine its impact on Mor-D/Mor-nD phenotypes. The potential link between social dominance and these phenotypes requires further investigation.

Together, our results provide novel information on the synaptic mechanisms that underlie negative affective states during opioid withdrawal. Our refined DBS treatment effectively prevented withdrawal symptoms, depressive phenotypes, and stress-induced reinstatement of morphine in mice. These findings may lead to a promising DBS approach that could potentially be translated into clinical practice for opioid use disorder.

## Methods

### Animals

Adult (8–10 weeks old) C57BL/6J male mice (Laboratory Animal Center of Xi’an Jiaotong University), Drd1-Cre and Drd2-eGFP mice (Institute of Health Sciences, Shanghai Institute of Life Science, Chinese Academy of Science), Drd1-tdTomato mice (The Jackson Laboratory), Drd1-tdTomato×Drd2-eGFP double-transgenic mice, and KOR^loxP/loxP^ mice (Cyagen, Suzhou, China) were used in this study. In our experiments, mice were kept in a temperature- and humidity-controlled environment with a 12-hour light/12-hour dark cycle (lights on at 7 am). All mice were acclimated to housing conditions for about 2 weeks (3 per cage, food and water ad libitum) before any experimental manipulations. All mice received treatments in the same order.

### Chronic morphine treatments

HCl-morphine (Third Research Institute of the Ministry of Public Security, Shanghai, China) and naloxone (N822820, MACKLIN) were prepared in 0.9% sodium chloride. Chronic morphine treatment was performed as previously described (17). Mice were injected i.p. twice daily with saline or escalating doses of morphine from 10 to 50 mg/kg during 5 days. On the morning of the sixth day, the last single dose of morphine (50 mg/kg) was administered.

### Naloxone-precipitated withdrawal

To measure withdrawal symptoms, withdrawal was precipitated by naloxone (1 mg/kg, s.c.) administration 2 hours after the last morphine injection (50). The withdrawal symptoms were monitored immediately for 20 minutes after naloxone injection (3, 51).

Each animal was scored individually (see Supplemental Methods). The number of wet dog shakes, front paw tremors, scratches, jumps, and sniffing episodes was counted. Body tremor, ptosis, teeth chattering, and piloerection were scored 1 for appearance or 0 for no appearance within 5-minute bins. A global withdrawal score was calculated for each animal by giving each withdrawal symptom a relative weight: 0.5 for each episode of wet dog shake, paw tremor, scratching, sniffing, and jumping; and 1 for presence of body tremor, ptosis, mastication, and piloerection during each 5-minute observation period.

### Conditioned place preference

During the preconditioning phase, mice that spent more than 600 seconds of a 15-minute period in either chamber or crossed between chambers fewer than 20 times were excluded from analysis. On the test day, mice were allowed to freely explore chambers for 15 minutes without injections. The time spent in each chamber was determined using a video tracking system.

During extinction training, all mice were given saline (10 mL/kg, i.p.) once daily and were immediately confined to chambers for 30 minutes on alternate days. After 8 days of extinction training, place preference was tested by allowing mice to freely explore conditioned place preference (CPP) chambers for 15 minutes. The mice that showed CPP extinction were subjected to stress-induced reinstatement. Morphine- and saline-trained extinguished animals were exposed to a single 20 minutes of foot shock stress session (Supplemental Methods). Immediately after foot shock, CPP was again determined. On the following day, animals were challenged with a single dose of morphine (10 mg/kg, i.p.) and allowed to explore all chambers for 15 minutes. As an index of general opioid effects, mice were weighed daily during chronic morphine injections and CPP and after 1 or 4 weeks of spontaneous withdrawal (i.e., in the absence of naloxone injection). Twenty-four hours and 1 and 4 weeks after the last injection, emotional-like responses were tested in the following order: sucrose preference test (SPT), open-field test (OFT), elevated plus maze (EPM), tail suspension test (TST), and forced swim test (FST) (Supplemental Figure 1).

### Behavioral testing

Behavioral tests were performed as described by Cerniauskas et al. (52) with minor modifications.

#### SPT.

The SPT procedure has been widely used to evaluate depressive-like behaviors (53, 54). The SPT assesses an animal’s preference for a sweet solution (1% sucrose dissolved in water) relative to plain water, and failure to have a preference indicates anhedonia, a core symptom of depression. During adaptation, mice were habituated to single housing in the same cages and given 24 hours of free access to 2 identical bottles containing water. Then, one of the drinking bottles was randomly exchanged with a bottle containing 1% sucrose solution for 24 hours. Before the testing day, mice were deprived of water for 23 hours. After deprivation, mice were presented with 2 drinking bottles for 1 hour (9 to 10 am); one bottle contained water and the other contained 1% sucrose solution. To eliminate the impact of preference in side, the bottles’ locations were randomly assigned and alternated at 12 hours (adaptation) or 30 minutes (testing). Bottles were weighed before and after testing session, and sucrose preference was defined as (sucrose solution intake)/(total fluid intake) × 100%.

#### TST.

Mice were suspended by the tail 50 cm above floor. The activity was automatically monitored during the last 4 minutes of the 6-minute test with a threshold defining immobility behavior. Latency to the first immobilization was also recorded.

#### FST.

Mice were placed for 6 minutes in a glass cylinder (height, 27 cm; diameter, 18 cm) filled with 3.5 liters of water (24°C ± 2°C), and immobility time was automatically monitored during the last 4 minutes of the 6-minute test. Latency to the first immobilization was also recorded.

#### NSF.

The novelty-suppressed feeding (NSF) test has been used for depression-related assessment. The NSF test was performed in a plastic box (50 × 50 × 20 cm). The floor was covered with approximately 2 cm of bedding, and the arena was brightly lit (1,100–1,200 lux). Mice were food-restricted for 24 hours. At time of testing, a single pellet of food was placed on a white paper platform positioned in the center of the box. Each animal was placed in a corner of the box. The amount of time to take the first bite was recorded as latency to feed. Home cage food consumption was measured right after the test as a control value.

### Deep brain stimulation

For all surgeries, anesthesia was induced with 5% isoflurane and maintained at 2%–3% (wt/vol) for duration of surgery. Polyamide-insulated stainless steel monopolar electrodes (125 μm in diameter, with 0.2 mm of surface exposed) were bilaterally implanted into the anterior pole of NAc (anterior-posterior [AP] 1.4 mm, medial-lateral [ML] ±1.0 mm, dorsal-ventral [DV] –4.5 mm). Anodes were connected to a screw on the skull, and an additional 2 screws were used to secure the implant. DBS was applied for 1 hour using a portable stimulator set to deliver 50 μA current (biphasic pulses, 90-microsecond pulse width). A stimulation frequency of 130 Hz or 50 Hz was used. DBS was applied 24 hours before behavioral testing. These settings were used to approximate a charge density similar to that used with humans, and set below induction of motor effects as previously described (55). Harvested brains were sectioned coronally (20 μm) on a Leica CM-3000 cryostat (Leica Microsystems) microtome at –20°C and thaw-mounted onto Fisher Scientific Positive Charge glass microscope slides. Slides were post-fixed in 10% formalin vapor, stained with cresyl violet, and then examined at ×10 magnification under a microscope.

### Stereotaxic viral injection

During surgery, mice were fixed in a stereotaxic apparatus (RWD Life Science) under isoflurane anesthesia. The coordinates used were as follows: NAc (AP 1.4 mm, ML ±2.0 mm, DV –4.6 mm, 10° angle), PVT (AP –1.5 mm, ML 0.5 mm, DV –3.2 mm, 10° angle), BLA (AP –1.4 mm, ML ±3.3 mm, DV –4.6 mm). A volume of 200–300 nL virus was infused bilaterally into the target brain region using a calibrated glass microelectrode attached to a 1 μL Hamilton microsyringe at a flow rate of 30 nL/min (Supplemental Table 1). The injector was left in place for an additional 15 minutes to minimize diffusion up the injector tract. After surgery, mice were singly housed for 48 hours and then placed back in their home cage.

### Intracerebral cannulation

Under isoflurane anesthesia and craniotomy as described above, steel guide cannulae (26 G) were implanted bilaterally in NAc (AP +1.4 mm, ML ±1.0 mm, DV –3.7 mm). Three screws were fastened into the skull before the implant. After cannulae were inserted, the cannulae and screws were cemented in place with dental cement. Fifteen to thirty minutes before behavioral procedures, mice were given 200 nL intracranial injections of either 0.9% isotonic saline or clozapine *N*-oxide (CNO) (300 mM; C0832, MilliporeSigma) at a rate of 100 nL/min.

### Slice electrophysiology

#### Slice preparation.

Animals were anesthetized by i.p. injection of sodium pentobarbital (90 mg/kg). Coronal 300-μm-thick slices of mouse brain were prepared with a vibratome (Leica VT1000S) in an ice-cold NMDG-based solution: 92 mM *N*-methyl-d-glucamine (NMDG), 2.5 mM KCl, 1.20 mM NaH_4_PO_4_, 30 mM NaHCO_3_, 20 mM HEPES, 25 mM glucose, 2 mM thiourea, 5 mM Na-ascorbate, 3 mM Na-pyruvate, 0.5 mM CaCl_2_, and 10 mM MgSO_4_ (300 mOsm, 7.2–7.4 pH). Slices were then allowed to recover at 34°C for 15 minutes in the same NMDG-based solution, and subsequently at room temperature, in carbonated regular artificial cerebrospinal fluid (aCSF): 119 mM NaCl, 2.5 mM KCl, 1.2 mM NaH_2_PO_4_, 24 mM NaHCO_3_, 12.5 mM glucose, 2 mM MgSO_4_, 2 mM CaCl_2_ (300 mOsm, 7.2–7.4 pH). Following at least 1 hour of recovery, slices were transferred to a recording chamber perfused with regular aCSF delivered at 2 mL/min at 30°C–32°C.

#### Whole-cell patch-clamp recordings.

The whole-cell voltage-clamp recordings were performed as previously described (54). The recordings were obtained from MSNs of NAc shell under visual guidance by differential interference contrast fluorescent microscopy (BX61WI, Olympus; fluorescent light U-RFL-T). Cells were identified based on visualization of presence of eGFP or tdTomato fluorophore. Borosilicate glass recording microelectrodes (World Precision Instruments) were pulled (resistance 4–6 MΩ) on a P-97 horizontal puller (Sutter Instruments) and backfilled with internal solution containing 102 mM cesium gluconate, 103 mM CsOH, 5 mM QX-314-Cl, 0.2 mM EGTA, 5 mM TEA-Cl, 20 mM HEPES, 2.8 mM NaCl, 4 mM Mg-ATP, 0.3 mM Na-GTP, and 10 mM Na-phosphocreatine. Recordings were initiated after seal rupture, initial stabilization, and equilibration of the whole-cell configuration for at least 3 minutes to allow dialysis of internal recording solution. The holding potential was –70 mV, and access resistance was monitored by a hyperpolarizing step of –5 mV with each sweep, every 10  seconds. Liquid junction potentials were left uncompensated. Signals were recorded using a Multiclamp 700B amplifier (Molecular Devices) or using an IPA-2 integrated patch amplifier controlled with SutterPatch software (Sutter Instruments). Only cells with a stable access resistance less than 25 MΩ throughout recording period were included in analysis.

To calculate input-specific A/N ratio, ChR2-mediated synaptic currents were optogenetically evoked by 473 nm blue light (4-millisecond pulse duration, at 0.1 Hz) through a ×40 objective at an intensity that evoked approximately 50% maximum responses. The evoked responses were measured at –70 mV (AMPAR-only response) and +40 mV (AMPAR and NMDAR combined response). Six traces, collected at 20-second intervals, were averaged at each holding potential. The A/N ratio was calculated by division of the amplitude of AMPAR-only response at +40 (the component within a 1-millisecond window of –70 mV peak) by the amplitude of NMDAR-only response measured in a 10-millisecond window beginning 50 milliseconds after stimulation.

To induce input-specific LTP at D1 MSNs, Chronos-mediated optogenetic high-frequency stimulation (oHFS; 473 nm, 100 pulses at 50 Hz, 4 trains of stimuli repeated at an interval of 20 seconds) was applied with ChrimsonR-mediated optogenetic postsynaptic depolarization (oPSD; 590 nm, 2-second pulse duration) through the objective. D1 MSNs were marked with a fluorescent mCherry tag due to the Cre-dependent action of ChrimsonR-mCherry, while the MSNs that lacked the fluorescent tag were identified as D2 MSNs (10, 28, 29). To induce input-specific long-term depression (LTD) at D2 MSNs, ChR2-mediated low-frequency stimulation (473 nm, 1 Hz for 10 minutes) was delivered with 4-millisecond light pulses. D2 MSNs were marked with D2-eGFP fluorescent tag, whereas the MSNs that lacked the fluorescent tag were identified as D1 MSNs. The magnitude of LTP or LTD was determined by comparison of average EPSCs recorded 20–25 minutes after induction with average EPSCs recorded 0–5 minutes before induction. These experiments were performed in the presence of picrotoxin (100 μM).

For recordings of cellular E/I balance, the intracellular recording solution contained 103 mM d-gluconic acid instead of cesium gluconate. Synaptic currents were electrically evoked by stimuli (50–100 microseconds) at 0.1 Hz through a bipolar stainless steel electrode placed onto tissue. EPSC was recorded at –60 mV (reversal potential for inhibitory responses), and inhibitory postsynaptic current (IPSC) was recorded at 0 mV (reversal potential for excitatory responses) with the NMDAR blocker AP5 (100 μM, bath application). Five responses at each holding potential were triggered and averaged. The E/I ratio was calculated using the average amplitudes of electrical EPSC (eEPSC) and eIPSC.

### Immunofluorescence histochemistry

Mice were perfused with cold 0.01 M of sodium phosphate-buffered 0.9% (wt/vol) saline (PBS, pH 7.4) and 4% (wt/vol) paraformaldehyde in 0.1 M of phosphate buffer (PB; pH 7.4). After perfusion, brains were further post-fixed in 4% paraformaldehyde overnight. Cryoprotected with 30% (wt/vol) sucrose in 0.1 M of PB for 24 hours, the whole brains were serially cut into 30-μm-thick transverse sections with a freezing microtome (CM1950, Leica). The sections were rinsed in 0.01 M PBS 3 times and blocked in 0.01 M PBS containing 10% normal donkey serum and 0.3% (vol/vol) Triton X-100 for 1 hour at room temperature. The blocked sections were then incubated overnight at room temperature with a mixture of rabbit anti-FG (1:1,000; AB153-I, Millipore) and mouse anti–c-Fos (1:200; ab208942, Abcam) in PBS containing 0.3% (vol/vol) Triton X-100, 0.25% (wt/vol) λ-carrageenan, and 5% (vol/vol) donkey serum (PBS-XCD). Sections were incubated at room temperature with Alexa Fluor 488–conjugated donkey anti-rabbit IgG (1:500; A21206, Invitrogen) for 6 hours and Alexa Fluor 594–conjugated donkey anti-mouse IgG (1:500; A11055, Invitrogen) for 10 minutes. The sections were observed with a confocal laser scanning microscope (FV-1000, Olympus). Confocal images were obtained, and digital images were captured using a Fluoview laser scanning confocal microscope (Olympus) equipped with FV1000 (version 1.7a) software.

### Fluorescence in situ hybridization

We synthesized digoxigenin-labeled (DIG-labeled) antisense single-strand RNA probes of KOR (http://mouse.brain-map.org) with a DIG RNA labeling kit (11277073910, Roche Diagnostics). Target sections were treated with 2% H_2_O_2_ in 0.1 M of diethyl pyrocarbonate–PB for 10 minutes. After rinsing with 0.1 M diethyl pyrocarbonate–PB and reacting in acetylation solution, the sections were pre-hybridized for 1 hour at 58°C in hybridization buffer. Then 1 μg/mL KOR RNA probe was added and hybridized at 58°C for 20 hours. After rinsing in wash buffer for 20 minutes twice at 58°C, the hybridized sections were incubated with 20 μg/mL ribonuclease A for 30 minutes at 37°C. The sections were incubated overnight with 0.5 μg/mL peroxidase-conjugated anti-DIG sheep antibody (11-207-733-910, Roche Diagnostics). We performed biotinylated tyramine–glucose oxidase amplification to amplify KOR hybridization signals. The sections were subsequently treated with 10 μg/mL Fluorescein Avidin D (A-2001; Vector Laboratories) for 4 hours. Then the sections were observed with a confocal microscope.

### Immunoblotting

The NAc tissues were processed for protein extraction as previously described (56). Protein samples were separated by 12% SDS-PAGE and transferred onto PVDF membranes. The membranes were blocked with 5% bull serum albumin and then incubated with primary antibodies (MOR, ab10275, Abcam; DOR, ab176324, Abcam; KOR, ab183825, Abcam) overnight at 4°C. Then, the membranes were washed and incubated with HRP-conjugated secondary antibodies. An enhanced chemiluminescence kit (WBKLS0100, Millipore) was used to detect immunoreactive protein bands. Band intensities were analyzed with ImageLab software (version 5.2.1, Bio-Rad). Protein expression levels were normalized to GAPDH expression levels, and data were presented as relative quantifications.

### Statistics

To determine whether individual animals were positive or negative for a specific behavioral phenotype as measured in SPT, TST, and FST, we used ROC curves (57). Youden J index was calculated from ROC curves to identify the optimal cutoff value that gave the lowest false positive rate (FPR) and the highest true positive rate (TPR). Youden J index maximizes difference between TPR (sensitivity) and FPR (1 − specificity): Youden index = TPR − FPR = sensitivity + specificity − 1. Thus, by maximizing of sensitivity + specificity across various cutoff points, the optimal cutoff point was calculated.

To determine statistical differences for histological, behavioral, and electrophysiological data, we performed Mann-Whitney *U* test, Student’s *t* test (paired and unpaired), and 1- and 2-way ANOVA (ordinary and repeated measures) using GraphPad Prism 8.0 software. Kruskal-Wallis, Tukey’s, or Šidák’s post hoc analysis was applied, when applicable, to correct for multiple comparisons. *P* values less than 0.05 were considered statistically significant (*P < 0.05, **P < 0.01, ***P < 0.0001). All data are presented as mean ± SEM. Investigators were blinded to allocation of groups and outcome assessment for all experiments.

### Study approval

The Institutional Animal Care and Use Committee of Xi’an Jiaotong University approved all procedures. All participants provided written informed consent. The Ethical Committee of the Medical College, Xi’an Jiaotong University, approved the study protocol.

### Data availability

Data are available from the corresponding authors JL or YW upon request. Values for all data points in graphs can be found in the Supplemental Supporting Data Values file.

## Author contributions

YW and JL contributed to the experimental design, data analysis, and writing of the manuscript. YZ, KW, YJ, Y Lou, XF, Y Lu, Y Liu, WE, QZ, FY, KW, BY, and HZ performed the behavioral and molecular experiments. TM performed the electrophysiological experiments.

## Supplementary Material

Supplemental data

Supporting data values

## Version 1 08/10/2023

In-Press Preview

## Version 2 09/15/2023

Electronic publication
