# Supplementary material for: Nucleus accumbens D1/D2 circuits control opioid withdrawal symptoms in mice
Source: J Clin Invest. 2023 Sep 15;133(18):e163266. doi: 10.1172/JCI163266 (PMC10503809; doi:10.1172/JCI163266)
Supplement: Supplemental data [file jci-133-163266-s106.pdf]

SUPPLEMENTAL METHODS

Subjects

Men were selected because they comprise 88.3% of the population of drug abusers in China (1). Subject were 576 heroin abusers in Xi'an Lantian and Zhouzhi Compulsory Isolated Detoxification Center (Xi'an, China). Participants were included if they (i) were daily or nearly daily users of heroin for a minimum of one year prior to assessment, (ii) were more than one month in drug withdrawal, (iii) never received any antidepressant, antipsychotic drug or mood stabilizer for at least two weeks prior to the study, and (iv) never received methadone maintenance treatment (MTT). The Observation Withdrawal Syndrome Scale used in the present study was provided by Xi'an Mental Health Center (Xi'an, China) (Table S4). The overall scores including 4 grades are 52. The major depression (MD) was diagnosed during structured clinical interviews based on the DSM-V criteria for MD. Depressive symptoms were rated by the 17-item Hamilton Depression Rating Scale (HAM-D, MD$\geq$17). The classification of the patients with major depressive disorders was based on total score (Table S5) (2). We did not attempt to distinguish between patients with heroin-induced depression and primary depressive disorders. All interviewers were trained before they entered the study. Participants were excluded if they had a history of amphetamine, ketamine, barbiturate, benzodiazepine, marijuana, or other drug addiction (nicotine and alcohol) according to DSM-V, bipolar disorder, schizophrenia, or were taking other prescribed medications that could affect the central nervous system. All participants were self-identified as Han Chinese for three generations. All participants provided written

informed consent. The Ethical Committee of the Medical College, Xi'an Jiaotong University approved the study protocol.

Somatic signs of morphine withdrawal

To measure physical dependence, withdrawal was precipitated by naloxone (1 mg/kg), administered either 2 h after the last morphine injection (3). Somatic signs of withdrawal were evaluated immediately after antagonist injection during a period of 20 min in a 50 cm high, 30 cm diameter transparent cylinder, put on white filter paper, under a 50 Lux indirect lighting. Each animal was scored individually. The number of wet dog shakes, front paw tremors, scratches, jumps and sniffing episodes was counted. Body tremor, ptosis, teeth chattering, and piloerection were scored 1 for appearance or 0 for nonappearance within 5 min bins. A global withdrawal score was calculated for each animal by giving each somatic sign a relative weight: 1 for each episode of wet dog shake, paw tremor, scratching, sniffing and jumping; and 1 for the presence of body tremor, ptosis, teeth chattering and piloerection during each observation period of 5 min (summed maximal score foe each sign is 4). A global withdrawal score was calculated as (wet dog shakes + front paw tremors + scratches + jumps + sniffing) × 0.5 + (body tremor + ptosis + teeth chattering + piloerection) × 1.

Behavior test

Footshock. Mice were shuttled from the CPP testing room to an intentionally different adjacent room with the shock apparatus, put in the shock box for 5 min of habituation, and then exposed to 15 min of random shocks (0.8 mA) that lasted 0.5 s each with an inter-shock interval from 10 to 70 s (mean of 40 s) (4).

OFT. Mice were placed during 15 min in 45×45×30 cm open-field arenas under a 150 Lux indirect lighting. The arena was divided into a central zone (40% of the total area) and a peripheral zone (60% of the total area) (5). Total distance and time spent in the center were automatically monitored and analyzed by Anymaze video-tracking software (Ver. 5.2, Stoelting).

EPM. Mice were placed in the center zone (6.5× 6.5 cm), facing a same open arm of the elevated plus maze (elevated 54 cm above the floor) with two open arms (30-cm length, 7-cm width) and two wall-enclosed arms (closed arms, 30-cm length, 6-cm width, walls 14.5 cm high) and allowed to explore freely for 5 min (6). Their path was video-tracked using Anymaze software, and the amount of time spent and entries in the open arms, closed arms, and center zone were recorded.

Reference:

1. Sun HQ, Bao YP, Zhou SJ, Meng SQ, and Lu L. The new pattern of drug abuse in China. *Curr Opin Psychiatry.* 2014;27(4):251-5.
2. Hamilton M. A rating scale for depression. *J Neurol Neurosurg Psychiatry.* 1960;23:56-62.
3. Contet C, Filliol D, Matifas A, and Kieffer BL. Morphine-induced analgesic tolerance, locomotor sensitization and physical dependence do not require modification of mu opioid receptor, cdk5 and adenylate cyclase activity. *Neuropharmacology.* 2008;54(3):475-86.
4. Nygard SK, Hourguettes NJ, Sobczak GG, Carlezon WA, and Bruchas MR. Stress-Induced Reinstatement of Nicotine Preference Requires Dynorphin/Kappa Opioid Activity in the Basolateral Amygdala. *J Neurosci.* 2016;36(38):9937-48.
5. Prut L, and Belzung C. The open field as a paradigm to measure the effects of drugs on anxiety-like behaviors: a review. *Eur J Pharmacol.* 2003;463(1-3):3-33.
6. Groessl F, Munsch T, Meis S, Griessner J, Kaczanowska J, Pliota P, et al. Dorsal tegmental dopamine neurons gate associative learning of fear. *Nat Neurosci.* 2018;21(7):952-62.

SUPPLEMENTAL FIGURES

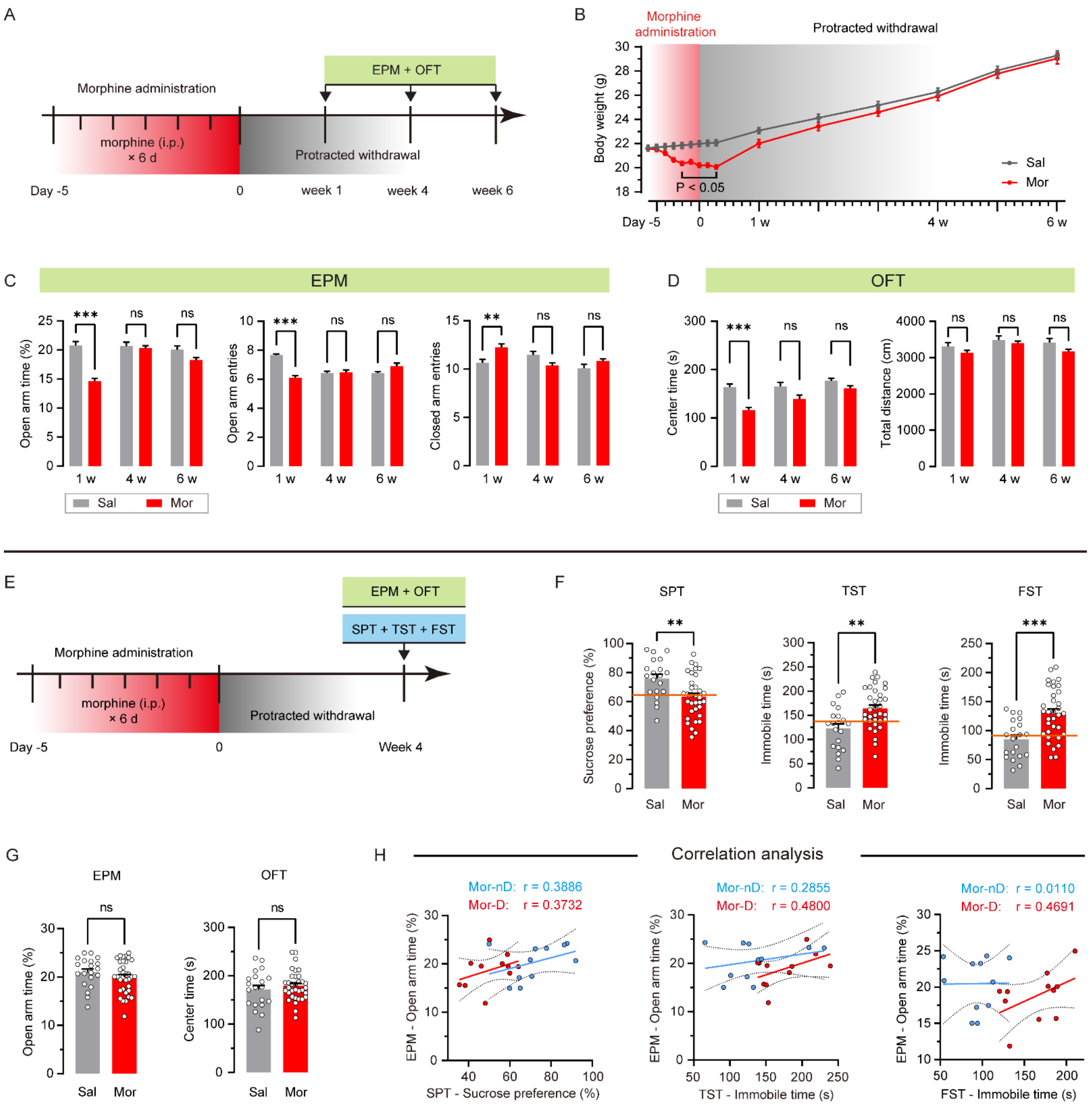

Figure S1. The anxiety-like behaviors test in morphine-withdrawal mice.

(A) Flow chart of evaluating anxiety-like behaviors. **(B)** Body weight during protracted withdrawal. **(C)** Anxiety-like behaviors in EPM. **(D)** Behaviors in OFT. **(E)** Flow chart of anxiety-like and depressive-like behaviors after protracted withdrawal. **(F)** Depressive-like behaviors. **(G)** Anxiety-like behaviors. **(H)** Correlation between depressive- and anxiety-like behaviors. Data are presented as mean ± SEM. $^{**}P < 0.01$, $^{***}P < 0.0001$, compared to Sal controls or the indicated group.

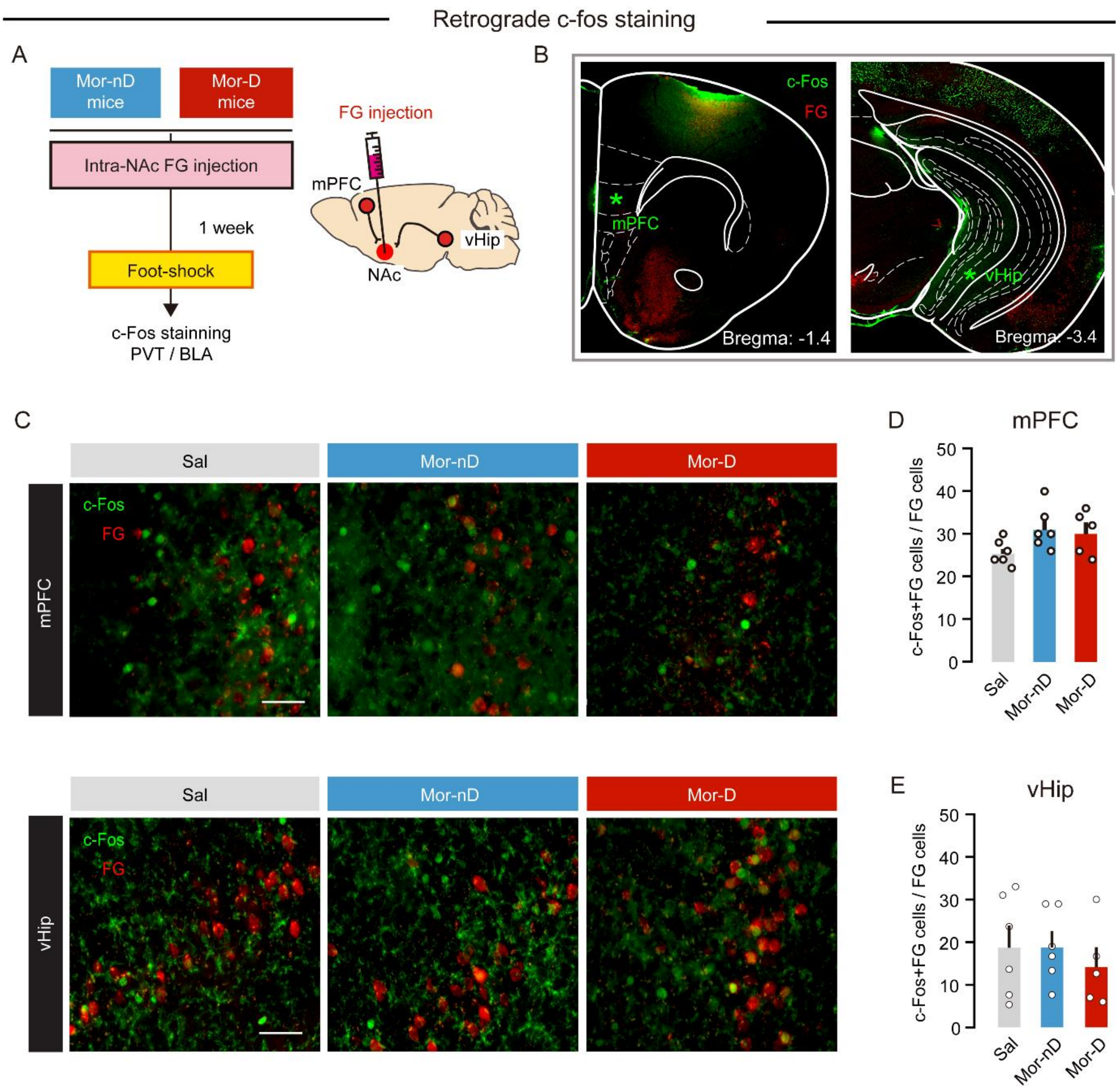

Figure S2. The mPFC and vHip afferents to NAc were not activated in Mor-D mice. **(A)** Experimental design for retrograde c-Fos staining. The retrograde tracer fluorogold (FG) was injected into NAc. c-Fos staining was performed in mPFC and vHip. **(B)** Representative photos of the co-labeling of c-Fos and FG in mPFC and vHip. **(C)** Co-labeling of c-Fos and FG in mPFC and vHip of Sal, Mor-nD and Mor-D mice (4 slices/mouse, n = 5-6/group). Scale bar = 100 μm. **(D)** Active c-Fos cells (c-Fos+FG / FG cells) in mPFC and **(E)** vHip. Data are presented as mean ± SEM.

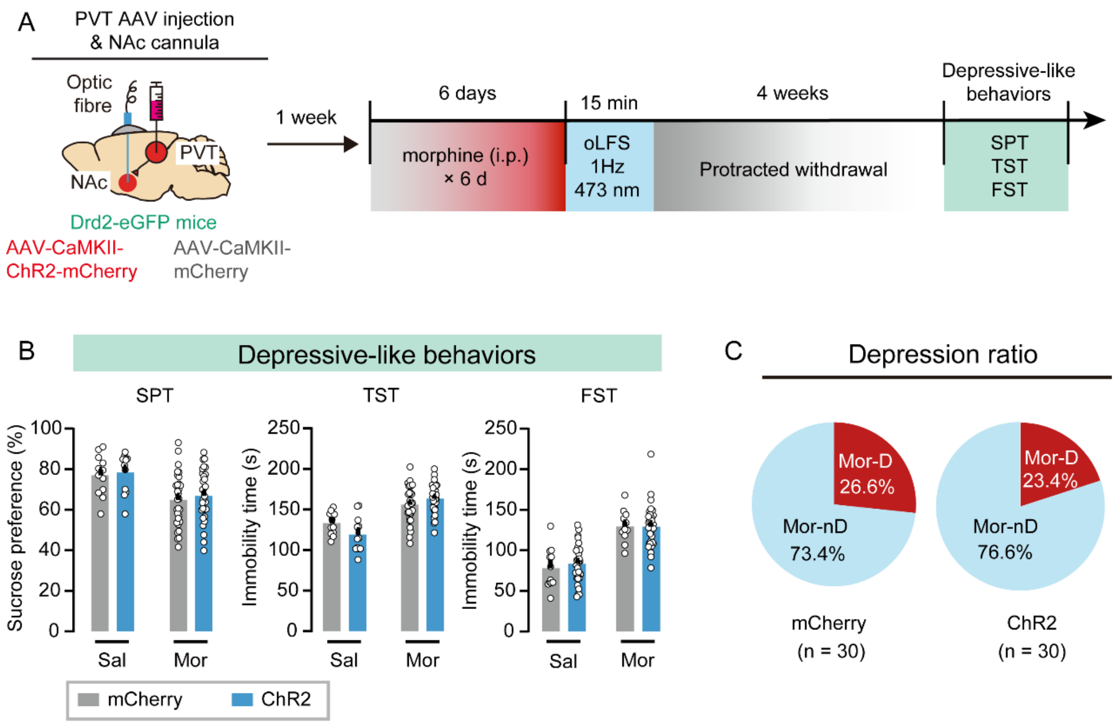

Figure S3. The depressive-like behaviors test in Mor-D mice after oLFS *in vivo*.

(A) Schematic and flow chart of *in vivo* optogenetic depotentiation of the PVT→NAc^{D2} pathway. **(B)** Depressive-like behaviors. **(C)** Depression ratios. Both the ChR2 and the mCherry groups showed a similar incidence of depressive-like behaviors. Data are presented as mean ± SEM.

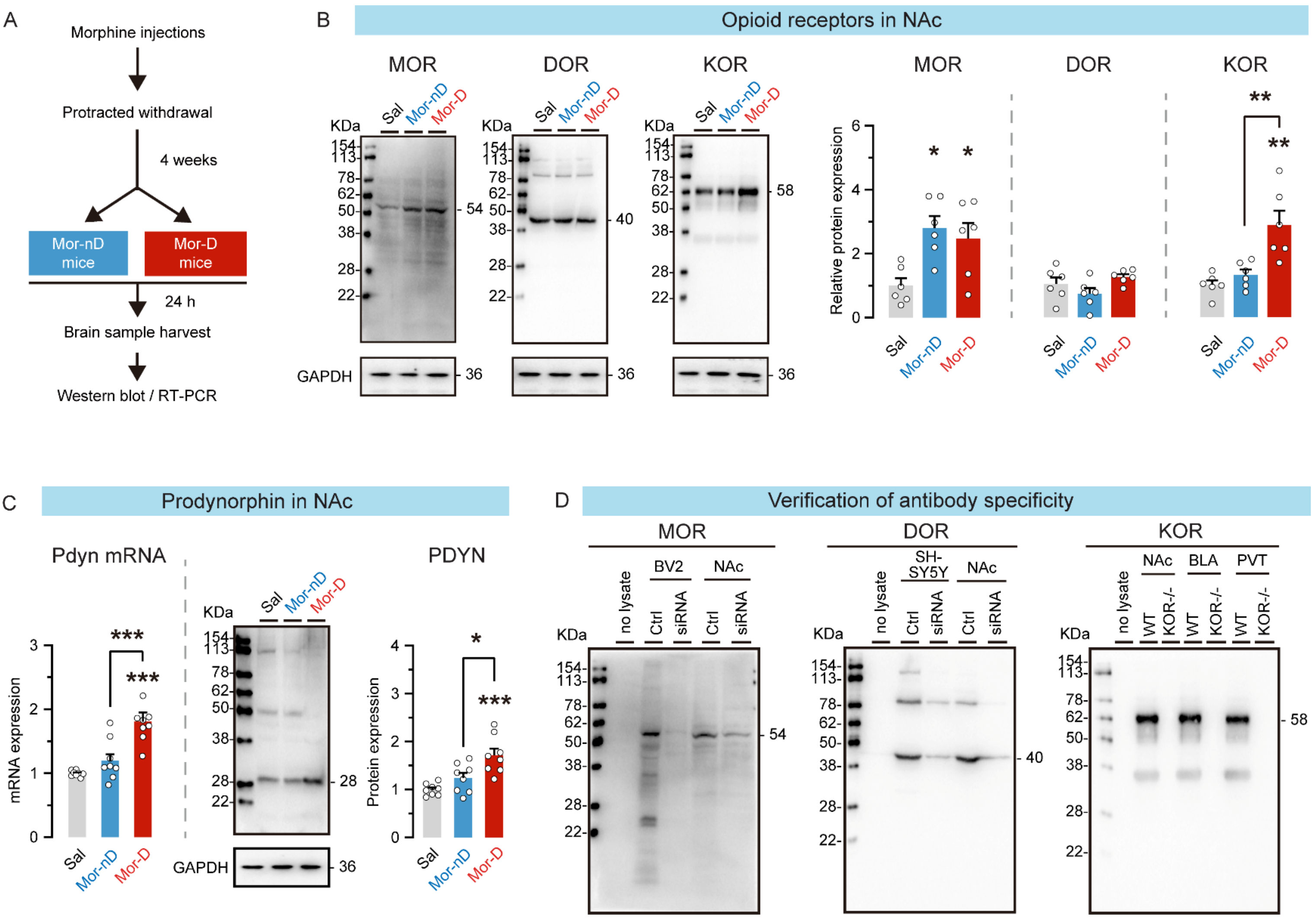

Figure S4. Immunoblots of the opioid receptor and PDYN in NAc.

(A) Flow chart of the opioid receptors expression test in NAc. **(B)** Expression level of mu (MOR), delta (DOR), and kappa (KOR) opioid receptors in NAc (n = 6/group). **(C)** *Left*: Representative immunoblots of prodynorphin (PDYN) in NAc. *Right*: Expression level of PDYN in NAc (n = 6/group). **(D)** Validation of the specificity of the antibodies for MOR, DOR, and KOR. Data are presented as mean ± SEM. $^{*}P < 0.05$, $^{**}P < 0.01$, $^{***}P < 0.0001$, compared with Sal controls or the indicated group.

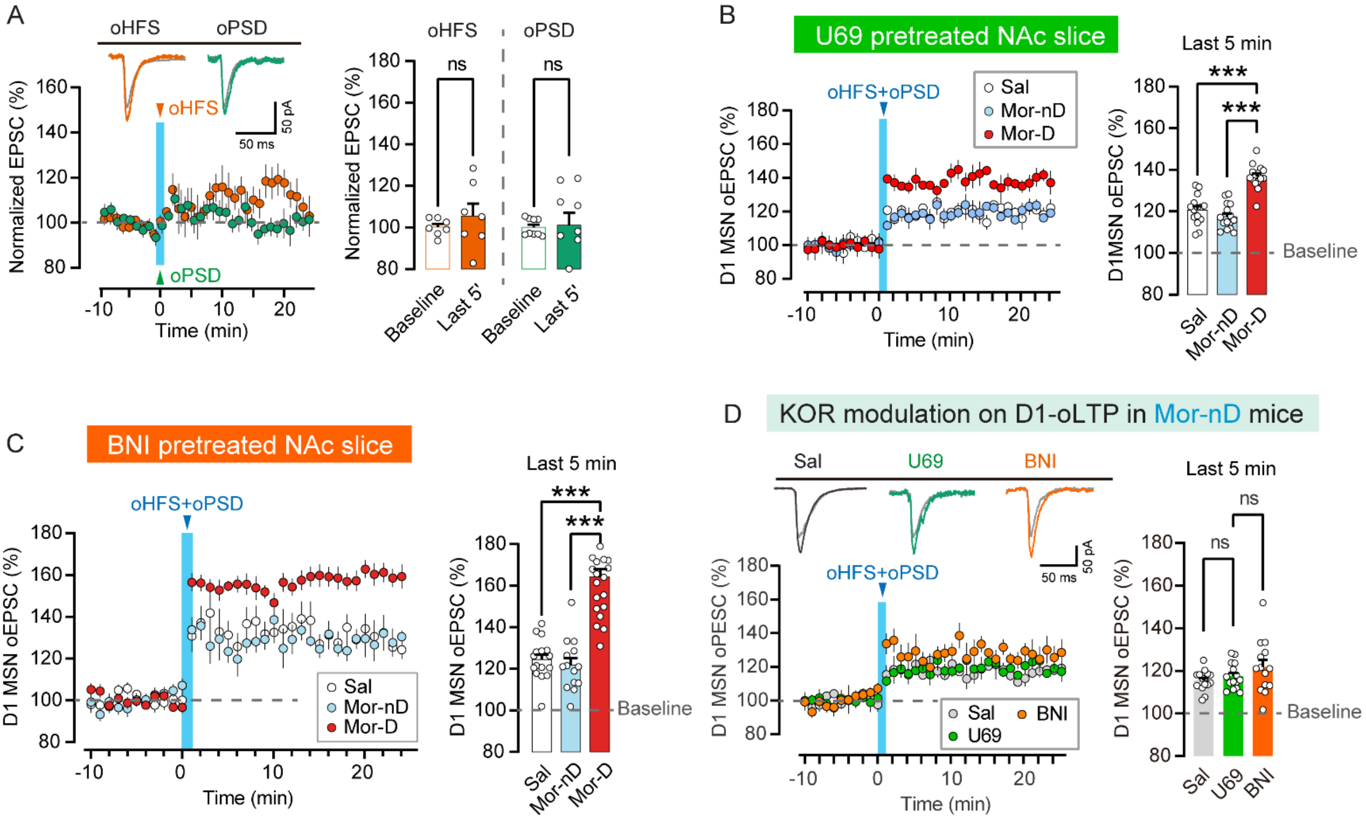

Figure S5. The optogenetic-induced EPSC in BLA→NAc^{D1} pathway.

(A) oEPSC was not changed in D1 MSN when only optical high-frequency stimulation (oHFS) or optical postsynaptic depression (oPSD) were applied. n = 7 neurons from 2 animals. **(B)** oEPSC increased (136.8±1.52% to the baseline) in D1 MSN in Mor-D mice from U69-pretreated NAc slices. n = 15 neurons from 3 animals per group. **(C)** oEPSC increased to a greater amplitude (164.3±3.65% to the baseline) in D1 MSN in Mor-D mice from BNI-pretreated NAc slices. n = 14-22 neurons from 3-6 animals per group. **(D)** oEPSC in D1 MSN of Mor-nD mice was not changed by pretreatment with BNI or U69. n = 14-15 neurons from 3-5 animals per group. Data are presented as means±SEM. $^{***}P < 0.0001$, compared with Sal controls or the indicated group.

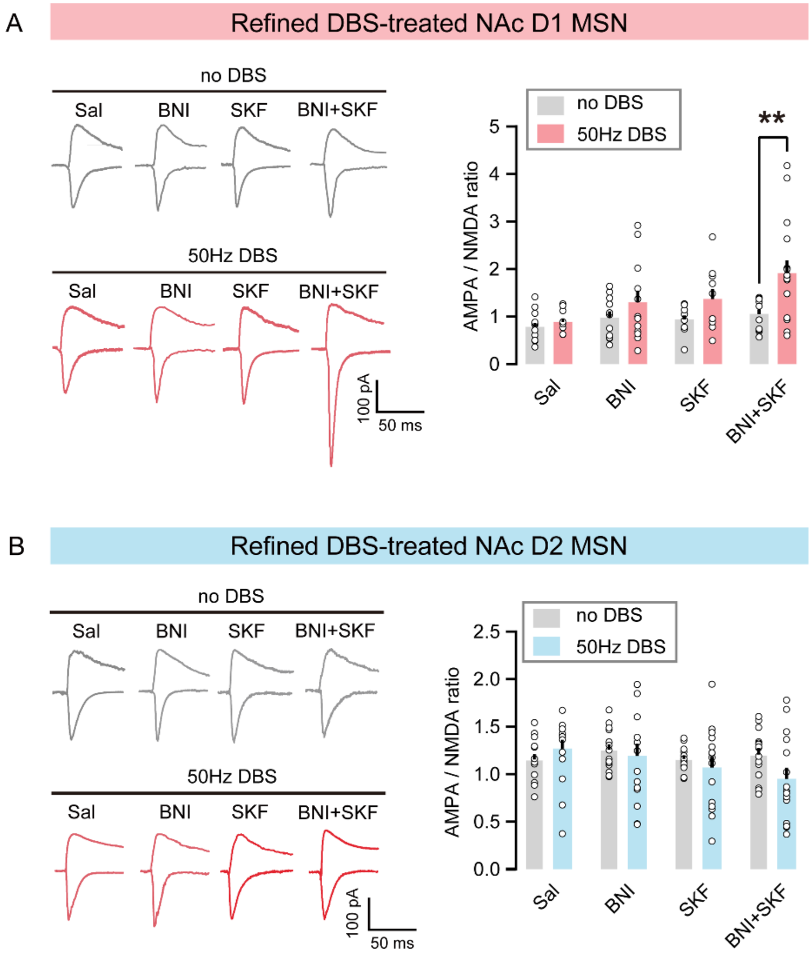

Figure S6. KOR blockade with DBS+SKF selectively elevated A/N ratio in NAc D1 but not D2 MSN.

(A) AMPA/NMDA (A/N) ratio in D1 MSN when treated with BNI, SKF or 50 Hz of DBS. n = 10-16 neurons from 3-5 animals per group. (B) AMPA/NMDA (A/N) ratio in D2 MSN. n = 15 neurons from 3-4 animals per group. Data are presented as mean ± SEM. **P < 0.01, compared with noDBS group.

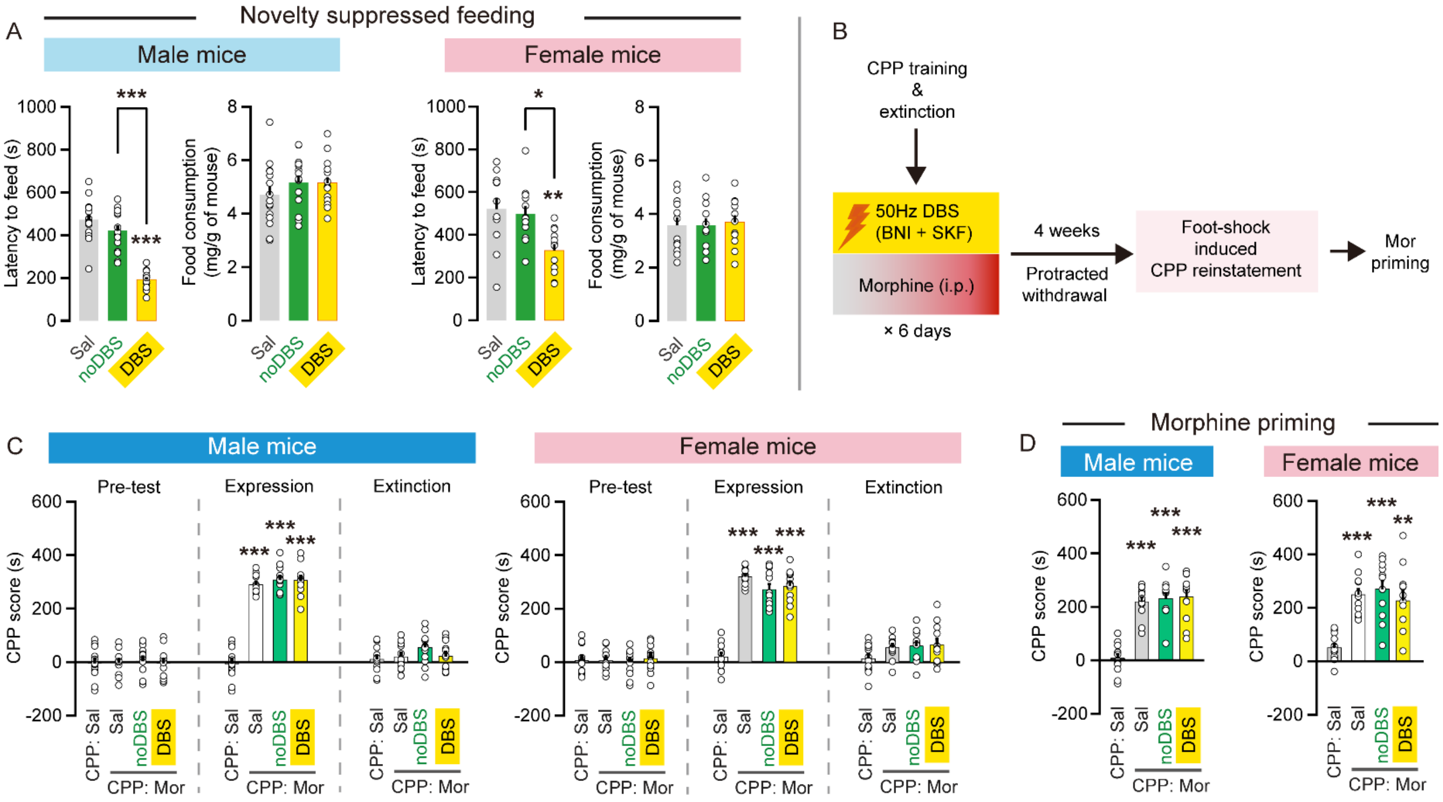

Figure S7. The novelty suppressed feeding and the CPP test in different stages of male and female mice.

(A) Novelty suppressed feeding (NSF) test in male and female mice. (B) Flow chart of CPP tests at different stages. After the extinction of morphine CPP, mice were treated with refined DBS (50 Hz DBS in NAc plus systemic administration of norBNI and SKF38393) for 6 days. After a protracted withdrawal period, mice were subjected to foot-shock induce CPP reinstatement and morphine induced CPP reinstatement. (C) Morphine CPP score in the pre-test, expression, and extinction stage. (D) CPP reinstatement induced by a priming dose of morphine (10 mg/kg, i.p.). Data are presented as mean ± SEM. *P < 0.05, **P < 0.01, ***P < 0.0001, compared to Sal controls or the indicated group.

SUPPLEMENTAL TABLES

Table S1. The virus was infused bilaterally into the target brain regions

	BLA	PVT	NAc
Fig. 2F	rAAV2/9-CaMKII-ChR2-mCherry	rAAV2/9-CaMKII-ChR2-mCherry	
Fig. 3A	pAAV2/9-CaMKII-Chronos-EGFP		rAAV2/9-hSyn-DIO-ChrimsonR-mCherry
Fig. 3H	pAAV2/9-CaMKII-Chronos-EGFP		rAAV2/9-hSyn-DIO-chrimsonR-mCherry
Fig. 3K	rAAV2/9-CaMKII-DIO-hM4D(Gi)-mCherry		rAAV2/retro-hSyn-cre-EGFP
Fig. S6A		rAAV2/9-CaMKII-ChR2-mCherry	
Fig. S6D		rAAV2/9-CaMKII-ChR2-EGFP	

Table S2. Somatic withdrawal signs following the naloxone-precipitated withdrawal and 4-week of spontaneous withdrawal from morphine treatment

	Signs	Saline (n = 12)	Morphine (n = 30)	*P* values	Significant?
Naloxone-precipitated withdrawal	Wet dog shakes	0.58 ± 0.19	3.47 ± 0.43	0.0002**	Yes
	Paw tremors	3.17 ± 0.32	36.7 ± 1.47	<0.0001***	Yes
	Scratching	4.17 ± 0.42	0.03 ± 0.02	<0.0001***	Yes
	Sniffing	0 ± 0	5.53 ± 0.5	<0.0001***	Yes
	Jumps	0 ± 0	38.93 ± 1.06	<0.0001***	Yes
	Body tremor	0.08 ± 0.05	2.3 ± 0.09	<0.0001***	Yes
	Ptosis	0 ± 0	1.07 ± 0.2	0.0017**	Yes
	Teeth chattering	0 ± 0	3.2 ± 0.22	<0.0001***	Yes
	Piloerection	0 ± 0	3.43 ± 0.17	<0.0001***	Yes
	GWS	4.04 ± 0.2	37.33 ± 1.13	<0.0001***	Yes
4 weeks of spontaneous withdrawal	Wet dog shakes	0.33 ± 0.19	0.43 ± 0.13	0.677	No
	Paw tremors	2.83 ± 0.28	1.83 ± 0.53	0.2529	No
	Scratching	3.83 ± 0.58	4.13 ± 0.31	0.6261	No
	Sniffing	0 ± 0	0.2 ± 0.17	0.4645	No
	Jumps	0 ± 0	0.1 ± 0.07	0.3752	No
	Body tremor	0.17 ± 0.11	0.06 ± 0.04	0.2457	No
	Ptosis	0 ± 0	0.17 ±0.12	0.3791	No
	Teeth chattering	0 ± 0	0 ± 0	N/A	N/A
	Piloerection	0.33 ± 0.26	0.16 ± 0.1	0.4577	No
	GWS	4 ± 0.32	3.78 ± 0.24	0.6124	No

Note: Two hours after the last saline or morphine injection, mice received a single sub-cutaneous naloxone injection (1 mg/kg) and were monitored for withdrawal signs. Withdrawal signs were also measured following 4 weeks of spontaneous abstinence. Withdrawal signs were individually observed and counted, and expressed as a global withdrawal score (GWS).

Table S3. Correlation coefficients across markers of depressive-like behaviors following morphine withdarwal

	**TST immobile time**	**FST immobile time**
**SPT sucrose preference**	-0.4537*	-0.47**
**TST immobile time**	/	0.4487*

Pearson's correlation: *p < 0.05, **p < 0.01.

Table S4. Persons with heroin addiction: Withdrawal Symptoms Scale

**Grade**	**Symptoms**	**Score**
I	A: Yawning B: Sweating C: Lacrimation D: Rhinorrhea E: Drowsiness	1 per item; total score: 5
II	A: Pupillary dilatation B: Gooseflesh C: Tremor D: Trembling E: Muscle aches and pains F：Anorexia	2 per item; total score: 12
III	A: Insomnia B: Hypertension C: Hyperpnea D: Tachycardia E: Restlessness F: Nausea	3 per item; total score: 15
IV	A: Curl-up position B: Vomiting C: Diarrhea D: weight loss E: Spontaneous ejaculation	4 per item; total score: 20

Table S5. Recommended ranges of scores on the 17-item Hamilton depression rating scale (HAMD) corresponding to levels of severity

**Depression severity**	**HAMD range of scores**
None	0–6
Moderate	7–17
Medium	18–24
Severe	≥24
